# The UBA1–STUB1 Axis Mediates Cancer Immune Escape and Resistance to Checkpoint Blockade

**DOI:** 10.1158/2159-8290.CD-24-0435

**Published:** 2024-11-14

**Authors:** Yi Bao, Gabriel Cruz, Yuping Zhang, Yuanyuan Qiao, Rahul Mannan, Jing Hu, Fan Yang, Mahnoor Gondal, Miriam Shahine, Sarah Kang, Somnath Mahapatra, Alec Chu, Jae Eun Choi, Jiali Yu, Heng Lin, Stephanie J. Miner, Dan R. Robinson, Yi-Mi Wu, Yang Zheng, Xuhong Cao, Fengyun Su, Rui Wang, Noshad Hosseini, Marcin Cieslik, Ilona Kryczek, Ulka Vaishampayan, Weiping Zou, Arul M. Chinnaiyan

**Affiliations:** 1 Michigan Center for Translational Pathology, University of Michigan, Ann Arbor, Michigan.; 2 Department of Pathology, University of Michigan, Ann Arbor, Michigan.; 3 Rogel Cancer Center, University of Michigan, Ann Arbor, Michigan.; 4 Department of Pathology, Qilu Hospital, Cheeloo College of Medicine, Shandong University, Jinan, China.; 5 Department of Computational Medicine and Bioinformatics, University of Michigan, Ann Arbor, Michigan.; 6 Department of Surgery, University of Michigan, Ann Arbor, Michigan.; 7 Center of Excellence for Cancer Immunology and Immunotherapy, University of Michigan, Ann Arbor, Michigan.; 8 Division of Hematology and Oncology, Department of Internal Medicine, University of Michigan, Ann Arbor, Michigan.; 9 Howard Hughes Medical Institute, University of Michigan, Ann Arbor, Michigan.; 10 Department of Urology, University of Michigan, Ann Arbor, Michigan.

## Abstract

How cancer cells escape immune surveillance and resist immune checkpoint blockade (ICB) remains to be fully elucidated. By screening candidate genes frequently gained in cancer, we identified expression of ubiquitin-like modifier-activating enzyme 1 (*UBA1*) as being the most negatively correlated with signatures related to effector CD8^+^ T cells. High *UBA1* expression was strongly predictive of treatment resistance and poor survival in ICB cohorts. Functional studies revealed that UBA1 mediated immune escape to promote tumor growth. Immune profiling further showed that *Uba1* overexpression or depletion markedly decreased or increased functional intratumoral CD8^+^ T cells, respectively. Importantly, a selective UBA1 inhibitor, TAK-243, significantly synergized with ICB in multiple syngeneic models. Mechanistically, depletion or inactivation of the UBA1–STUB1 axis stabilized a key IFN pathway component (JAK1), enhanced IFN signaling, and elevated key immune modulators, including CXCL9, CXCL10, and MHC class I. Our study warrants clinical evaluation of the combination of UBA1 inhibitors and ICB.

**Significance:**

Our study reveals UBA1 as a predictive biomarker for clinical outcomes in ICB cohorts, mediating cancer immune evasion and ICB resistance. We further highlight JAK1 stabilization as a key mechanism of UBA1 inhibition and nominate the UBA1–STUB1 axis as an immuno-oncology therapeutic target to improve the efficacy of ICB.

## Teaser

UBA1 is a potential cancer biomarker and therapeutic target that mediates immune evasion through JAK1 degradation, and targeted UBA1 inhibition synergizes with immune checkpoint blockade to enhance antitumor immunity and survival.

## Introduction

Cancer immunotherapies have achieved remarkable clinical success in certain indications (1–4). However, many patients fail to respond to these therapies, especially in cancer types considered immunologically “cold,” with fewer intratumoral immune effector cells present (5–8). There remains a critical need to define the mechanisms by which cancer cells evade immune surveillance and thus exhibit resistance to immunotherapies. Uncovering and targeting these mechanisms could greatly expand the pool of patients with cancer that benefit from immunotherapy.

Previous studies have associated genetic aberrations of IFN-γ pathway genes with primary or acquired resistance to immunotherapies (9–14). These aberrations include loss-of-function mutations in *JAK1* or *JAK2* (9); genomic loss of *IFNGR1*, *IRF1*, *JAK2*, and *IFNGR2* (10); and allelic loss of *JAK1* (9, 14). Importantly, in various preclinical settings, the loss of *JAK1*, *IFNGR1*, or *JAK2* has been found to confer resistance to immunotherapies (10, 15–17). However, homozygous loss or mutations of these genes are infrequent among nonresponders (12, 14), suggesting that tumor cells may adopt alternative mechanisms to inactivate or downregulate the products encoded by these genes.

Ubiquitin-like modifier-activating enzyme 1 (UBA1, also known as UBE1) is a primary E1 enzyme, at the apex of the ubiquitin-mediated proteasomal degradation machinery (18–20). UBA1 binds via ATP hydrolysis to and activates ubiquitin, which is then passed on to E2-conjugating enzymes (18) that are brought together with the protein targeted for ubiquitination by E3 ligases (18, 20). *UBA1* has been found to be essential in cancer cells (21). TAK-243 is a mechanism-based small-molecule inhibitor of UBA1 with selectivity over other E1 enzymes, including UBA6 (22, 23). Targeting UBA1 with TAK-243 has shown antitumor efficacy in various immunodeficient settings, and TAK-243 exhibited little or no toxicity in those preclinical models (22–25). Phase I clinical trials have been initiated to study TAK-243 in the treatment of cancer, including one which was terminated because of sponsor realignment of priorities (NCT02045095) and another which is ongoing (NCT03816319). Several mechanisms have been reported to explain the action of TAK-243 in tumors, including stabilization of tumor suppressors such as p53, leading to growth arrest and apoptosis (23); however, almost all preclinical studies testing TAK-243 were performed in immunodeficient models (23–25). Whether UBA1 functions in cancer immune evasion and whether UBA1 inhibition improves the efficacy of immunotherapies via eliciting antitumor immunity remain unexplored. Of note, somatic *UBA1* mutations have been attributed to an adult-onset inflammatory disorder, the VEXAS (vacuoles, E1 enzyme, X-linked, autoinflammatory, somatic) syndrome (26, 27), with elevation of canonical immune signatures, such as TNF, type-I IFN, and type-II IFN (26). This strongly implicates that modulation of UBA1 activity affects the expression of immune-related components.

Here, we report that elevated expression of *UBA1* is prevalent in cancer, associated with low levels of intratumoral CD8^+^ T cells, and predictive of immune checkpoint blockade (ICB) resistance and poor survival in ICB cohorts. Functionally, UBA1 mediates cancer immune evasion, and importantly, inhibition of UBA1 by TAK-243 markedly suppresses tumor growth in combination with ICB, exhibiting the potential of tumor clearance. Mechanistically, depletion or inactivation of the E1 enzyme UBA1 or the E3 ligase STUB1 stabilizes a key IFN pathway component, JAK1. Consequently, response to type-I and type-II IFNs is elevated, leading to increased expression of key immune modulators, including CXCL9, CXCL10, and MHC class I (MHC-I). Our study highlights that apart from genetic aberrations (9–13), key components in IFN signaling pathways can also be dysregulated by proteasomal degradation in cancer cells, leading to tumor progression. Our findings position UBA1 as a therapeutic target for activating anticancer immunity and improving the efficacy of ICB.

## Results

### High Expression of *UBA1* Is Associated with Low Levels of Intratumoral CD8^+^ T Cells

**Figure 1.**
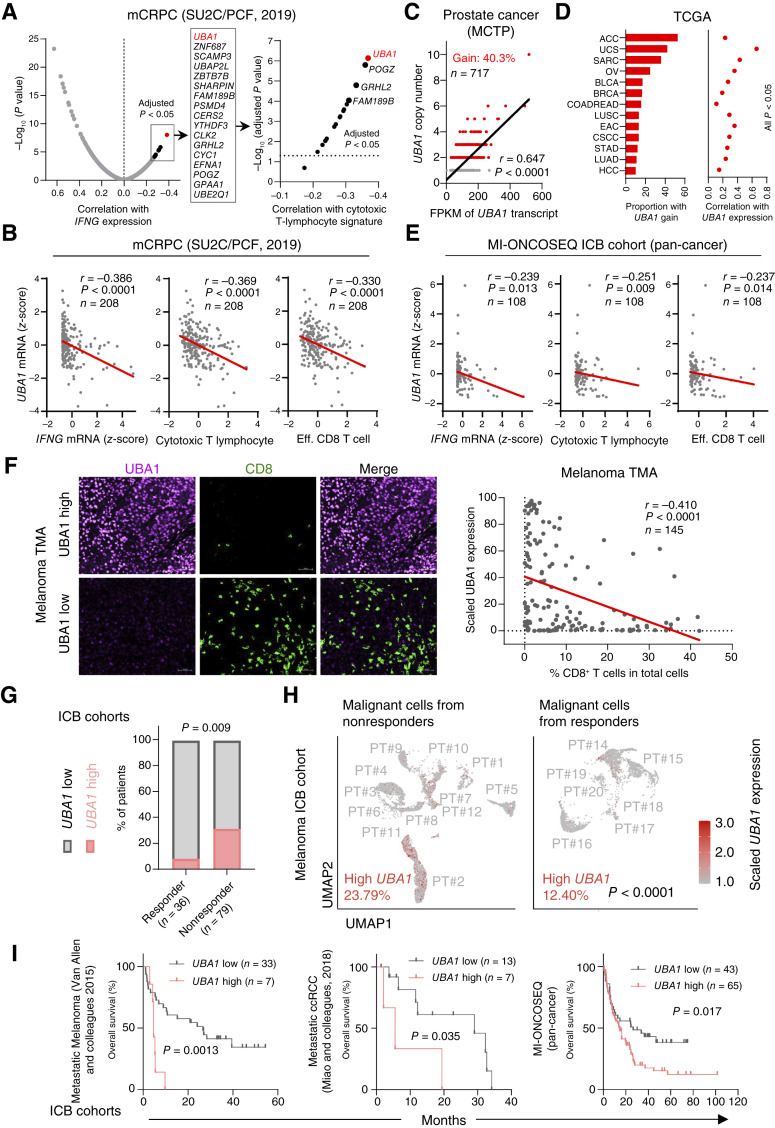
High expression of *UBA1* is associated with low levels of intratumoral CD8^+^ T cells and predictive of ICB resistance and poor survival in ICB cohorts. **A,** Left: Spearman correlation between mRNA expression of *IFNG* and 614 frequently gained genes in the indicated mCRPC cohort (*n* = 208). Genes that are significantly negatively correlated with *IFNG* mRNA expression are listed. Right: Spearman correlation between mRNA expression of the cytotoxic T-lymphocyte signature (*CD8A*, *CD8B*, *GZMA*, *GZMB*, and *PRF1*) and the genes listed on the left. SU2C, Stand Up to Cancer; PCF, Prostate Cancer Foundation. **B,** Spearman correlation between mRNA expression of *UBA1* and the indicated gene or gene signature in the indicated cohort. Eff., effector. **C,** Spearman correlation between *UBA1* copy number and mRNA expression in the indicated prostate cancer cohort. Frequency of copy number gain (gain) is shown. Patients with prostate cancer (male) with two or more copies of *UBA1* (on the X chromosome) are defined as gain. MCTP, The Michigan Center for Translational Pathology. **D,** Proportion of *UBA1* gain (left) and Spearman correlation between *UBA1* copy number and mRNA levels (right) in the indicated cancer types. Data were acquired from The Cancer Genome Atlas (TCGA). ACC, adrenocortical carcinoma; BLCA, bladder urothelial carcinoma; BRCA, breast invasive carcinoma; COADREAD, colorectal adenocarcinoma; CSCC, cervical squamous cell carcinoma; EAC, esophageal adenocarcinoma; HCC, hepatocellular carcinoma; LUAD, lung adenocarcinoma; LUSC, lung squamous cell carcinoma; OV, ovarian serous cystadenocarcinoma; SARC, sarcoma; STAD, stomach adenocarcinoma; UCS, uterine carcinosarcoma. **E,** Spearman correlation between pretreatment mRNA expression of *UBA1* and the indicated gene or signature, in a cohort treated with ICB at the U-M, Ann Arbor (MI-ONCOSEQ ICB cohort). **F,** Representative images (left) or quantification (Spearman correlation; right) of immunofluorescence assessing the number of CD8^+^ T cells and the level of UBA1 in a melanoma tissue microarray (TMA). Scale bar, 50 μm. **G,** Fisher exact test of a combined analysis on four public RNA-seq datasets [Van Allen and colleagues (34); Zhao and colleagues (35); Miao and colleagues (36); and Jung and colleagues (37)] examining the relationship between pretreatment mRNA expression of *UBA1* and response to ICB. **H,** Uniform Manifold Approximation and Projection (UMAP) of malignant cells from scRNA-seq data in an ICB-treated melanoma cohort. Cells with high levels of pretreatment *UBA1* mRNA expression are highlighted in pink. PT#, patient number. **I,** Overall survival of patients with tumors showing high or low pretreatment: *UBA1* mRNA levels in the indicated cohorts. ccRCC, clear-cell renal cell carcinoma. Statistics were acquired by two-tailed Student’s *t* test in **H**, and by log-rank test in **I**.

Prostate cancer has been considered immunologically “cold,” with relatively small fractions of patients responding to ICB (5, 6). In an effort to discover novel candidate proteins that may mediate immune evasion in cancer, we assessed the inverse correlation between expression of 614 frequently gained genes and *IFNG*, an antitumor gene expressed by immune effector cells, in 208 metastatic castration-resistant prostate cancer (mCRPC) samples [Fig. 1A (left)]. A total of 17 significant genes, the expressions of which were negatively correlated with *IFNG* expression, were identified [Fig. 1A (left)]. Among them, *UBA1* expression was the most negatively correlated with *IFNG* expression [Fig. 1A (left)]. The 17 candidates were further analyzed in correlation with the expression of a cytotoxic T-lymphocyte (CTL) signature (28), and *UBA1* expression was again the most negatively correlated [Fig. 1A (right)]. To examine how *UBA1* was ranked in the entire transcriptome, we correlated the levels of *IFNG* transcript with all other detected transcripts [total 19,007; Supplementary Fig. S1A (left)]. We found that *UBA1* was ranked within the top five percentile of transcripts that were negatively correlated with *IFNG* expression [Supplementary Fig. S1A (right)]. Of note, the top positively correlated list included genes expressed in functional CD8^+^ T cells [highlighted in blue in Supplementary Fig. S1A (left)], confirming the reliability of the dataset, whereas the top negatively correlated list included many genes that have been reported to mediate immune evasion or were associated with a cold tumor microenvironment [highlighted in pink in Supplementary Fig. S1A (right)]. Dot plots of individual cases further revealed that high-*UBA1*–expressing tumors were associated with low levels of *IFNG* or CTL signature [Fig. 1B (left and middle)], which was validated with another effector CD8^+^ T cell–related signature (Fig. 1B (right); refs. 29, 30].

In prostate cancer cohorts, *UBA1* exhibited frequencies of copy number gain higher than 40%, and importantly, the copy number of *UBA1* was strongly correlated with its mRNA expression (Fig. 1C; Supplementary Fig. S1B). Of note, *UBA1* was also frequently gained in many cancer types other than prostate cancer (Fig. 1D), whereas *UBA1* mutations in cancers were rare (Supplementary Fig. S1C). Importantly, in these cancer types, *UBA1* also exhibited positive correlations between DNA and mRNA levels (Fig. 1D). Concordantly, mRNA levels of *UBA1* were upregulated in various cancer types compared with the adjacent normal tissues (Supplementary Fig. S1D). A positive correlation between the copy number of *UBA1* and its mRNA expression implied that the *UBA1* gene was accessible in chromatin in cancer. In agreement with this, we found that hypermethylation on the *UBA1* promoter in cancer was rare (Supplementary Fig. S1E), in contrast to the reported (31–33) frequent hypermethylation on the *MGMT* promoter (Supplementary Fig. S1E). To confirm the upregulation of UBA1 at the protein level, we performed immunoblot analysis on the lysates from normal prostate tissues, primary prostate adenocarcinoma, and mCRPC (Supplementary Fig. S1F). We found that UBA1 was upregulated in 40% (2/5) of prostate adenocarcinomas and 60% (6/10) of mCRPCs (Supplementary Fig. S1F). Of note, the highly aggressive subtype of mCRPC, neuroendocrine mCRPC, showed the highest frequency of UBA1 upregulation (80%; 4/5; Supplementary Fig. S1F). These data, thus, show that UBA1 protein levels were upregulated during disease progression.

We next examined the correlation between expression of *UBA1* and *IFNG* or CD8^+^ T cell–related signatures in cancer types other than prostate cancer. We found that in both individual cancer (Supplementary Fig. S1G) and pan-cancer (Fig. 1E) cohorts, *UBA1* expression was significantly negatively correlated with the expression of *IFNG* or CD8^+^ T-cell–related signatures. These findings were further supported by histological staining in a pan-cancer cohort, whereby most tumors with high tumor-specific UBA1 expression were immune-cold with low amounts of CD8^+^ T cells, whereas a significant portion of low-UBA1–expressing tumors showed high levels of intratumoral CD8^+^ T cells (Supplementary Fig. S1H; Supplementary Table S1). Additionally, validation with a melanoma tissue microarray confirmed that protein levels of UBA1 were strongly negatively correlated with the abundance of intratumoral CD8^+^ T cells (Fig. 1F; Supplementary Fig. S1I).

### High Expression of *UBA1* Is Associated with ICB Resistance and Poor Survival in ICB Cohorts

We next examined whether the expression of *UBA1* was predictive of ICB response. With a combined analysis (*n* = 115) of four publicly available RNA sequencing (RNA-seq) datasets from various cancer types (34–37), we found that high pretreatment expression of *UBA1* was strongly predictive of ICB resistance (*P* = 0.009; Fig. 1G). We next analyzed a single-cell RNA-seq (scRNA-seq) dataset to determine whether high *UBA1* expression specifically in malignant cells was predictive of poor response to ICB. Indeed, we observed that ICB-nonresponsive patients exhibited a significantly higher proportion of high-*UBA1*–expressing malignant cells than responders (23.79% vs. 12.40%; Fig. 1H). We further determined the prognostic value of *UBA1* in ICB cohorts and found that high pretreatment *UBA1* expression was strongly associated with poor survival in both individual cancer and pan-cancer cohorts (Fig. 1I; Supplementary Fig. S1J). Collectively, these results identify *UBA1* as a new predictive biomarker of treatment resistance and poor survival in ICB cohorts.

### UBA1 Promotes Tumor Growth by Mediating Immune Escape

**Figure 2.**
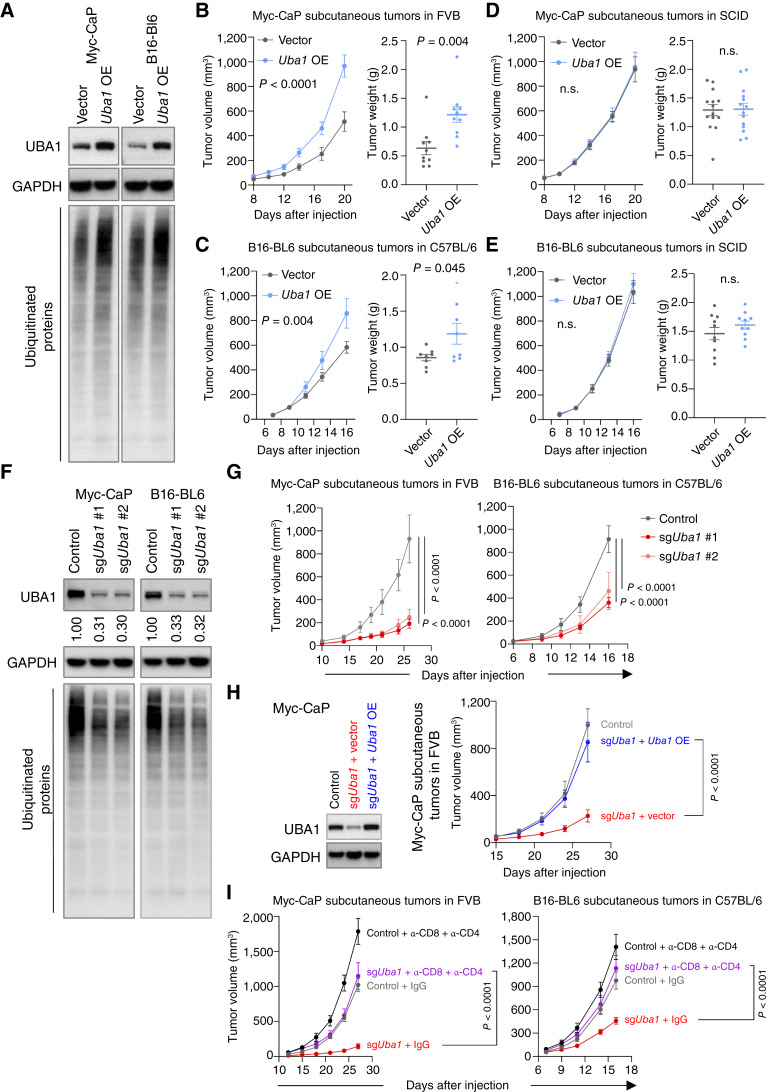
UBA1 promotes tumor growth by mediating immune escape. **A,** Immunoblot analysis assessing levels of the indicated proteins in the indicated cells transduced with empty vector or *Uba1* overexpression (OE). **B** and **C,** Volumes (left) and weights (right) of subcutaneous tumors derived from Myc-CaP (**B**) or B16-BL6 (**C**) cells established in **A**, in FVB or C57BL/6 mice, respectively (*n* = 5 mice per group in **B**; *n* = 4 mice per group in **C**). **D** and **E,** Volumes (left) and weights (right) of subcutaneous tumors established with injection of the indicated Myc-CaP (**D**) or B16-BL6 (**E**) cells to SCID mice (*n* = 7 mice per group in **D**; *n* = 5 mice per group in **E**). **F,** Immunoblot analysis assessing levels of the indicated proteins in the indicated cells transfected with nontargeting sgRNA (control) or independent sgRNAs depleting *Uba1* (sg*Uba1* #1 and sg*Uba1* #2). Quantification of intensity of UBA1 relative to the control is shown. **G,** Volumes of subcutaneous tumors derived from Myc-CaP (left) or B16-BL6 (right) cells established in **F**, in the indicated mice (*n* = 5–7 mice per group). **H,** Left: immunoblot analysis assessing *Uba1* overexpression (OE) in *Uba1*-depleted Myc-CaP. Right: volumes of subcutaneous tumors derived from Myc-CaP cells established in Left, in FVB mice (*n* = 5 mice, per group). **I,** Volumes of subcutaneous tumors established with injection of control or *Uba1*-depleted Myc-CaP (left) or B16-BL6 (right) cells to the indicated mice, with or without simultaneous depletion of both CD8^+^ and CD4^+^ T cells (*n* = 4–5, per group). Data are representative of two distinct sgRNAs. All data are presented as mean ± SEM. Statistics were acquired by two-way ANOVA in **B** (left), **C** (left), **D** (left), and **E** (left), and **G**–**I** (n.s., not significant), or by the two-tailed Student’s *t* test in **B** (right), **C** (right), **D** (right), and **E** (right). Data in **B**, **C**, and **G** are representative of two independent experiments.

As mentioned earlier, whether and how UBA1 functions in cancer progression or cancer immune evasion remains unaddressed. We thus established two murine syngeneic models, the prostate cancer Myc-CaP model and melanoma B16-BL6 model, with engineered overexpression of *Uba1* (Fig. 2A). These cells were injected into either syngeneic immunocompetent mice or immunodeficient SCID mice (Fig. 2B–E). *Uba1* overexpression significantly promoted tumor growth in the immunocompetent mice (Fig. 2B and C) but not in the immunodeficient mice (Fig. 2D and E). We also sought to deplete *Uba1* in these models with CRISPR (clustered regularly interspaced short palindromic repeats) knockout. In line with the notion that *UBA1* is an essential gene in cancer cells (21), we achieved partial depletion but not complete knockout of *Uba1* in these models (Fig. 2F; Supplementary Fig. S2A). Of note, both Myc-CaP (38) and B16-F0 (the parental cell line of B16-BL6; ref. 39) cells have been reported to carry two X chromosomes, which harbor the *Uba1* gene. This explains why partial depletion of *Uba1* could be achieved in these cells. We found that depletion of *Uba1* significantly decreased tumor growth in both syngeneic models (Fig. 2G; Supplementary Fig. S2B). Importantly, restoration of UBA1 reversed the tumor growth impairment caused by *Uba1* depletion, confirming that the CRISPR-mediated depletion of *Uba1* was on-target (Fig. 2H). To determine whether this antitumor effect was immune dependent, we depleted CD8^+^ T cells with an anti-CD8 antibody (Supplementary Fig. S2C) and observed that depletion of CD8^+^ T cells significantly, albeit partially, rescued the growth of *Uba1*-depleted tumors (Supplementary Fig. S2D), showing that CD8^+^ T cells were indispensable for the full tumor control mediated by *Uba1* depletion.

The partial rescue from CD8^+^ T cell depletion prompted us to deplete both CD4^+^ and CD8^+^ T cells, as CD4^+^ T cells might also mediate direct tumor control (40–42). We observed that simultaneous depletion of both CD4^+^ and CD8^+^ T cells resulted in a much stronger rescue of tumor growth in *Uba1*-depleted tumors (Fig. 2I). Consistently, although *Uba1* depletion caused elevation of both CD8^+^ and CD4^+^ intratumoral T cells, the levels of CD4^+^ T cells remained elevated after CD8^+^ T cell depletion (Supplementary Fig. S2E). By contrast, NK cell levels did not show a significant change in *Uba1*-depleted tumors compared with controls (Supplementary Fig. S2E).

Collectively, these data indicate that UBA1 plays a role in evading T-cell–mediated immune surveillance, thereby promoting tumor growth. In line with this, *Uba1* depletion only modestly affected cancer cell proliferation *in vitro* (Supplementary Fig. S2F).

### UBA1 Diminishes Intratumoral Effector CD8^+^ T Cell Levels

**Figure 3.**
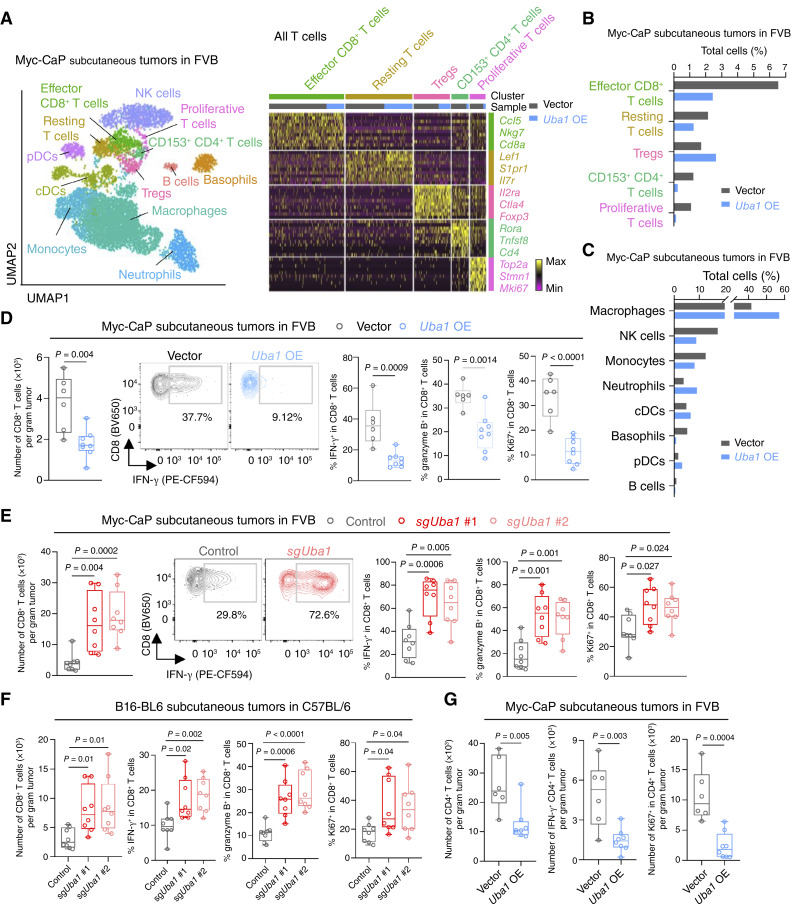
UBA1 diminishes intratumoral functional CD8^+^ T cells. **A,** Left: UMAP of 8,862 cells and the indicated clusters identified among CD45^+^ cells enriched from the indicated Myc-CaP tumors subjected to scRNA-seq. Right: heatmap showing differentially expressed genes in each of the indicated clusters among T cells. Three representative genes are shown on the right for each cluster. Proportions of T cells derived from the experimental group (*Uba1* overexpression) and control group (empty vector) are shown on the top. **B** and **C,** The fraction of each T-cell (**B**) or immune cell (**C**) subpopulation among all CD45^+^ immune cells from the indicated groups. **D** and **E,** Flow cytometry measuring the absolute numbers of CD8^+^ T cells (left) or proportions of IFN-γ^+^, granzyme B^+^, or Ki67^+^ cells among CD8^+^ T cells (right) in the indicated tumors. Middle: representative images showing the proportional change of IFN-γ^+^ CD8^+^ T cells by *Uba1* overexpression (**D**) or *Uba1* depletion (**E**). **F,** Flow cytometry measuring the absolute numbers of CD8^+^ T cells or proportions of IFN-γ^+^, granzyme B^+^, or Ki67^+^ cells among CD8^+^ T cells in the indicated tumors. **G,** Flow cytometry measuring the absolute numbers of CD4^+^ T cells, IFN-γ^+^ CD4^+^ T cells, or Ki67^+^ CD4^+^ T cells in the indicated tumors. All data are presented as box and whisker plots, except in **B** and **C** (bar graph). Statistics were acquired by the two-tailed Student *t* test. Data in **D**–**G** are pooled from two independent experiments.

We next sought to perform a comprehensive immune profiling in tumors with *Uba1* overexpression using scRNA-seq, identifying various immune cells (Fig. 3A; Supplementary Fig. S3A) and five subclusters of T cells (Fig. 3A). We found that compared with control tumors, *Uba1*-overexpressing tumors exhibited diminished proportions of effector CD8^+^ T cells, proliferative T cells, and a subset of CD4^+^ T cells expressing *Tnfsf8* (CD153; Fig. 3B). By contrast, the proportions of other immune cells that have established roles in antitumor immunity or protumor inflammation, such as NK cells, dendritic cells, Tregs, macrophages, and neutrophils, were less altered (Fig. 3C). These data support the notion that T cells, especially effector CD8^+^ T cells, were the major immune cells affected by the modulation of *Uba1* expression in tumor cells. We then performed flow cytometry analysis with a focus on CD8^+^ T cells (Supplementary Fig. S3B and S3C). In agreement with the scRNA-seq data, flow cytometry analysis revealed that the absolute amounts of CD8^+^ T cells were strikingly decreased in *Uba1*-overexpressing tumors compared with the control [Fig. 3D (left)]. Importantly, the proportions of CD8^+^ T cells expressing the functional markers, IFN-γ or granzyme B, or the proliferative marker Ki67, were also greatly reduced [Fig. 3D (middle and right)]. Conversely, in tumors with *Uba1* depletion, we observed significant increases of total, functional, and proliferative CD8^+^ T cells (Fig. 3E and F). The flow cytometry analysis also included CD4^+^ T cells (Supplementary Fig. S3B and S3C), and we found that the levels of total, IFN-γ^+^, and proliferative CD4^+^ T cells were significantly reduced in the *Uba1*-overexpressing tumors (Fig. 3G) and increased in *Uba1*-depleted tumors (Supplementary Fig. S3D). Of note, restoring UBA1 in *Uba1*-depleted tumors (Supplementary Fig. S2C) reversed their status from immune-hot to immune-cold, manifested by reduction of total, functional, and proliferative CD8^+^ and CD4^+^ T cells (Supplementary Fig. S3E). Taken together, the levels of T cells, especially functional CD8^+^ T cells, were greatly altered in tumors with modulated *Uba1* expression.

### UBA1 Inhibition Synergizes with Anti–PD-1 Therapy to Control Tumor Growth

**Figure 4.**
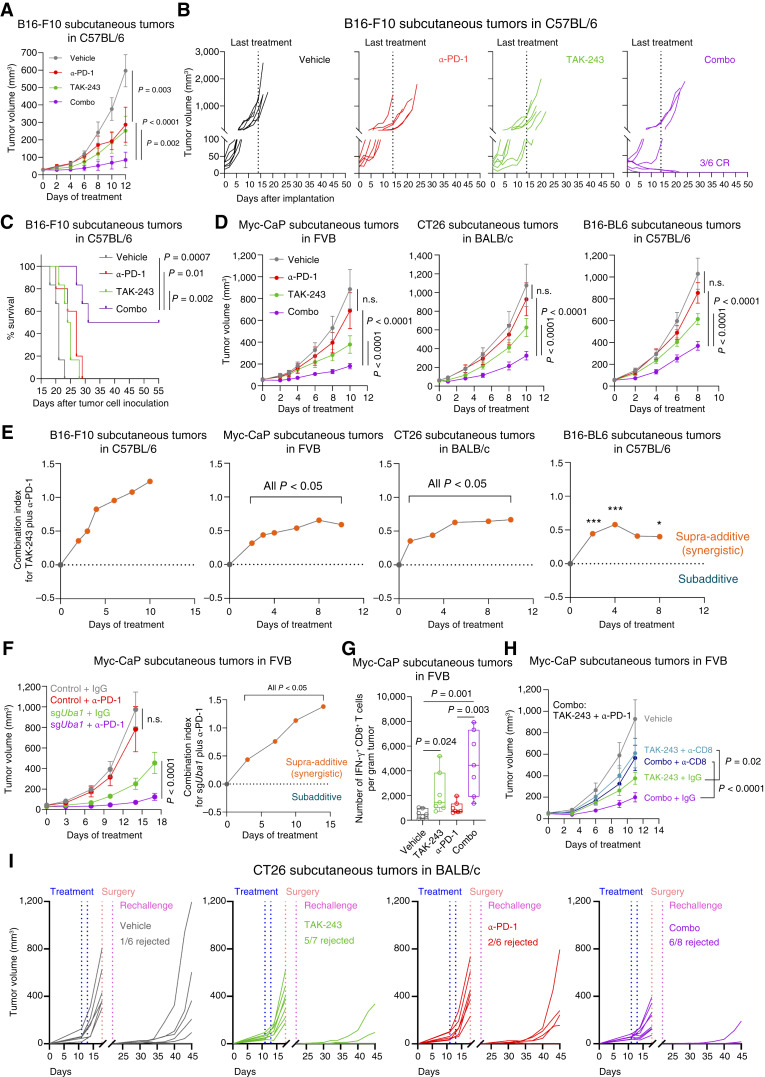
UBA1 inhibition synergizes with anti–PD-1 therapy to control tumor growth. **A,** Change of volume over time of subcutaneous tumors derived from B16-F10 cells in C57BL/6 mice treated with the indicated agents (*n* = 5–6 mice, per group). α-PD-1: anti–PD-1; Combo: TAK-243 plus α-PD-1. **B,** Individual growth curves of tumors in mice treated as in **A**. Last treatment was administered on day 14 after the initial treatment. CR, complete response. **C,** Survival of mice treated in **A**. **D,** Change of volume over time of the indicted tumor models in their syngeneic hosts treated with the indicated agents (*n* = 5–10 mice per group). **E,** Evaluation of drug synergism, using CombPDX (44), for the combination of TAK-243 and anti–PD-1 in the indicated models, treated as in **A** and **D**. A combination index larger than zero was defined as synergistic (44). *, *P* < 0.05; ***, *P* < 0.001. **F,** Left: change of volume over time of subcutaneous tumors derived from Myc-CaP cells transfected with nontargeting sgRNA (control) or sgRNAs depleting *Uba1* (sg*Uba1*) in FVB mice treated with α-PD-1 or the control IgG (*n* = 5 mice per group). Right: Evaluation of synergism using CombPDX for the combination of *Uba1*-depletion (sg*Uba1*) and anti-PD-1 in the indicated model. **G,** Flow cytometry measuring the absolute numbers of IFN-γ^+^ CD8^+^ T cells in the indicated tumors from syngeneic mice treated with the indicated agents. **H,** Tumor growth over time of Myc-CaP subcutaneous tumors in control, TAK-243–treated, or TAK-243–treated and anti-PD-1–treated (combo) FVB mice, with (α-CD8) or without (IgG) CD8^+^ T-cell depletion (*n* = 5 mice, per group). **I,** Tumor growth over time of CT26 subcutaneous tumors at naïve or rechallenged stage, in control, TAK-243–treated, anti–PD-1–treated, or TAK-243–treated and anti–PD-1–treated (combo) BALB/c mice. Rechallenge was performed 4 days after removal of the primary tumors by surgery (*n* = 6–8 mice, per group). All data are presented as mean ± SEM, except **E** (mean) and **G** (box and whisker plots). Statistics were acquired by two-way ANOVA in **A**, **D**, **F**, and **H**, by log-rank test in **C**, or by two-tailed Student’s *t* test in **G**.

Given the striking increase of CD8^+^ T cells in *Uba1*-depleted tumors, we sought to determine whether inactivation of UBA1 could affect response to ICB therapy. TAK-243 has been demonstrated to be a selective inhibitor of UBA1 (22, 23). In line with this, we observed a profound reduction of ubiquitinated proteins with TAK-243 treatment (Supplementary Fig. S4A). We next examined whether TAK-243 improved efficacy of anti–PD-1. In a melanoma tumor model B16-F10 that is modestly responsive to PD-1 blockade (Fig. 4A; ref. 43), we observed a striking combined effect of TAK-243 and anti–PD-1 (Fig. 4A), leading to tumor clearance in 50% (3/6) of mice (Fig. 4B). No tumors recurred in these mice after treatment was terminated when mice were followed through to the end of the experiment (55 days after tumor cell inoculation; Fig. 4B and C). By contrast, all mice in the vehicle group died or had to be euthanized within 23 days after tumor cell inoculation (Fig. 4C). This translated to a striking extension of survival in the combination-treated mice (Fig. 4C). Importantly, in models (prostate cancer Myc-CaP, colon carcinoma CT26, melanoma B16-BL6, and prostate cancer TRAMP-C2) that were insensitive to anti–PD-1 (Fig. 4D; Supplementary Fig. S4B), TAK-243 treatment significantly sensitized tumors to anti–PD-1 (Fig. 4D; Supplementary Fig. S4B). We also assessed the combination index for TAK-243 and anti–PD-1 with CombPDX, a tool developed for evaluating drug synergism *in vivo* (44). We found that, in all the models tested, TAK-243 was significantly synergistic with anti–PD-1 (Fig. 4E; Supplementary Fig. S4C). Of note, all animals tolerated TAK-243 or the combination treatment, exhibiting no noticeable body weight loss (Supplementary Fig. S4D), which is in line with the reported tolerance of TAK-243 in preclinical models (22, 23). Similarly, *Uba1* depletion improved efficacy of [Fig. 4F (left)] and synergized with [Fig. 4F (right)] anti–PD-1 to control tumor growth. Therefore, UBA1 inhibition or depletion augments response to anti–PD-1. Functional CD8^+^ T cells were upregulated in tumors from mice treated with TAK-243 or the combination (Fig. 4G), and importantly, CD8^+^ T cells were necessary for full tumor control mediated by TAK-243 or the combination (Fig. 4H).

As CD8^+^ T cells were involved in tumor control, we hypothesized that TAK-243 or the combination with anti–PD-1 could provide prolonged protection against cancer to the hosts. We thus established tumors in naïve mice and rechallenged the mice with tumor cell inoculation after two doses of vehicle, TAK-243, anti–PD-1, or the combination and surgical removal of initial tumors (Fig. 4I). We observed small fractions of tumor rejection (16.67%–33.33%; 1/6–2/6) in the rechallenged mice in the vehicle- and anti–PD-1–treated groups, showing establishment of immunologic memory, although TAK-243 or the combination with anti–PD-1 greatly increased the rejection percentages [71.43% (5/7) or 75% (6/8), respectively; Fig. 4I]. Moreover, tumors that outgrew in the rechallenged TAK-243– or combination-treated mice grew significantly slower than tumors in the vehicle- or anti–PD-1–treated mice, with the slowest growth rates observed in the combination group (Fig. 4I). As expected, tumors in the rechallenged TAK-243– or combination-treated mice exhibited higher amounts of memory CD8^+^ T cells (CD44^+^, CD62L^+^, and KLRG1^−^; Supplementary Fig. S4E; refs. 45, 46) than the vehicle- or anti–PD-1–treated mice (Supplementary Fig. S4F). Collectively, a systemic and prolonged protection against cancer was observed in the TAK-243– or combination-treated mice, accompanied with an increase of memory CD8^+^ T cells in tumors.

### UBA1 or STUB1 Inactivation Upregulates IFN Signaling via Stabilization of JAK1

**Figure 5.**
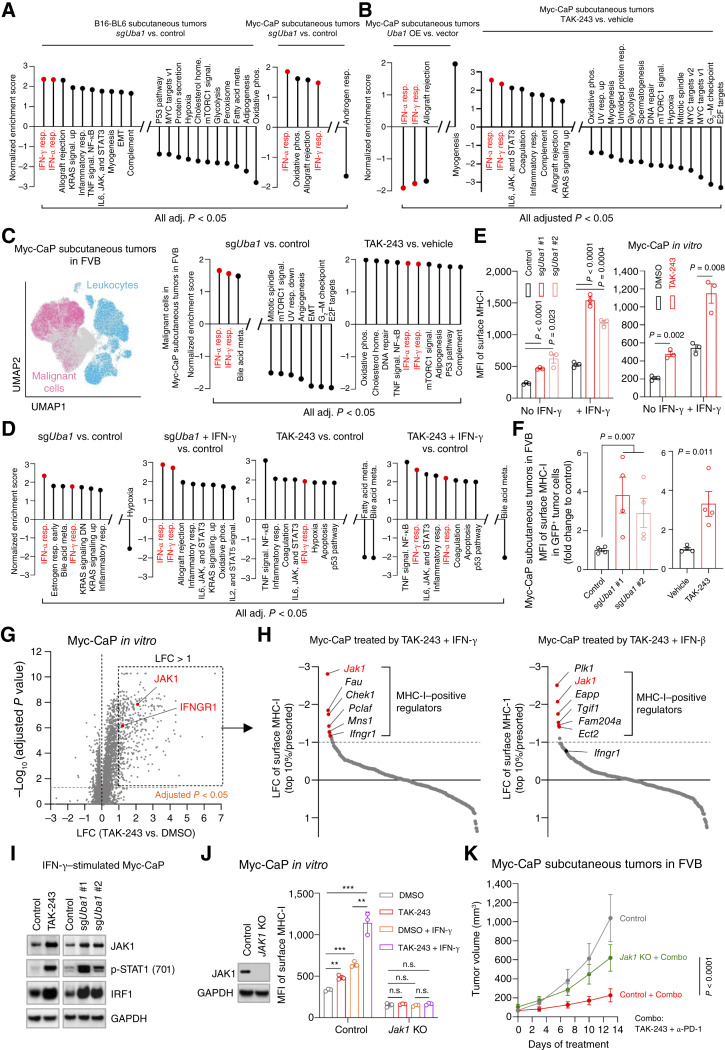
UBA1 inactivation upregulates interferon signaling via stabilization of JAK1. **A,** Hallmark pathways enriched by bulk RNA-seq of tumors with *Uba1* depletion (sg*Uba1*) versus control from the B16-BL6 (left) or Myc-CaP (right) subcutaneous tumor models. **B,** Hallmark pathways enriched by bulk RNA-seq of tumors with *Uba1* overexpression (OE) versus control (empty vector) from the Myc-CaP subcutaneous tumor model (left) or of Myc-CaP subcutaneous tumors in TAK-243–treated vs. control mice (right). **C,** Left: UMAP of pooled CD45^+^ and CD45^−^ cells from the indicated Myc-CaP tumors subjected to scRNA-seq. Clusters of malignant cells and leukocytes are shown. Right: hallmark pathways enriched by the scRNA-seq (shown in the left) of tumors with *Uba1* depletion (sg*Uba1*) versus control or tumors in TAK-243–treated vs. control mice. TAK-243 was administered via intravenous injection in **B** (right) and **C**. **D,** hallmark pathways enriched by bulk RNA-seq of Myc-CaP cells with *Uba1* depletion (sg*Uba1*) vs. control, with or without IFN-γ stimulation, or Myc-CaP cells treated with or without 50 nmol/L TAK-243 for 18 hours, and stimulated with or without IFN-γ. IFN-γ or IFN-α response pathways are highlighted in red in **A**–**D**. **E,** Surface expression of MHC-I measured by flow cytometry in Myc-CaP cells with *Uba1* depletion (sg*Uba1*) or UBA1 inhibition by 18 hours of 50 nmol/L TAK-243 treatment, in the presence or absence of IFN-γ stimulation. Nontargeting sgRNA or DMSO were used as controls, respectively. Data were acquired from biological triplicates. **F,** Surface expression of MHC-I measured by flow cytometry in GFP-labeled Myc-CaP tumor cells that were *Uba1* depleted or inactivated (*n* = 4 mice, per group). **G,** Mass spectrometry measuring protein abundance in Myc-CaP cells treated with 100 nmol/L TAK-243 for 4 hours and subsequently 50 µg/mL of cycloheximide (CHX) for an additional 6 hours. LFC, Log_2_ fold change. **H,** CRISPR knockout screens with sgRNAs targeting genes that were robustly upregulated by TAK-243 in **G**, in Myc-CaP cells that received TAK-243 and IFN-γ co-treatment (left) or TAK-243 and IFN-β co-treatment (right). **I,** Immunoblot analysis assessing levels of the indicated proteins in Myc-CaP cells with *Uba1* depletion (sg*Uba1*) or 18 hours of 50 nmol/L TAK-243 treatment in the presence of IFN-γ stimulation. Nontargeting sgRNA or DMSO were used as controls, respectively. **J,** Left: immunoblot analysis assessing JAK1 expression in Myc-CaP cells that received knockout of *Jak1* (*Jak1* KO). Cells receiving nontargeting sgRNA were used as control. Right: surface expression of MHC-I measured by flow cytometry in the indicated cells treated with or without 50 nmol/L TAK-243 and stimulated with or without IFN-γ. Data were acquired from technical triplicates, representative of two independent experiments. **K,** Volumes of tumors derived from Myc-CaP cells established as in **J**, in mice treated with or without the combination (combo) of anti-PD-1 and TAK-243 (*n* = 5 mice, per group). Data in **J** and **K** are representative of two independent experiments with two distinct sgRNAs. IFN-γ stimulation was performed at 1 ng/mL for 18 hours. All immunoblot analysis was representative of two independent experiments. Data are presented as mean ± SEM. Statistics were acquired by the two-tailed Student *t* test in **E**, **F** (TAK-243 vs. DMSO), and **J**, or by two-way ANOVA in **F** (sg*Uba1* vs. control) and **K**. **, *P* < 0.01; ***, *P* < 0.001; n.s., not significant. MFI, mean fluorescent index.

To determine the mechanism by which modulation of *Uba1* expression affected tumor growth in a CD8^+^ T cell–dependent manner, we performed RNA-seq analysis on tumors with *Uba1* depletion or overexpression and found that both type-I and type-II IFN signaling pathways were among the most deregulated pathways in tumors with modulated *Uba1* expression (Fig. 5A and B; Supplementary Fig. S5A and S5B). Specifically, these pathways were upregulated in tumors with *Uba1* depletion (Fig. 5A; Supplementary Fig. S5A) and downregulated in tumors with *Uba1* overexpression [Fig. 5B (left); Supplementary Fig. S5B]. Consistently, tumors derived from the TAK-243–treated mice also showed upregulation of the pathways compared with the control [Fig. 5B (right); Supplementary Fig. S5C]. Using scRNA-seq that distinguished malignant cells from other cells [Fig. 5C (left)], we further specified that the IFN pathways [Fig. 5C (right)] and IFN-regulated genes (Supplementary Fig. S5D) were upregulated in malignant cells in *Uba1*-depleted tumors or tumors from TAK-243–treated mice. Intriguingly, significant enrichment of these pathways was observed in cancer cells with *Uba1* depletion or TAK-243 treatment *in vitro*, particularly in the presence of IFN-γ stimulation (Fig. 5D; Supplementary Fig. S5E). Analysis of the differentially expressed genes revealed that expression of a set of IFN-γ–regulated genes (47) were strikingly upregulated under IFN-γ stimulation in the TAK-243–treated cells compared with the control (Supplementary Fig. S5F). These genes included key immune modulators, such as *Cxcl9* and *Cxcl10*, responsible for recruiting CD8^+^ T cells (48–50), and *H2-K1* and *H2-D1*, encoding MHC class I (MHC-I) genes crucial for antigen presentation and thus tumor recognition by CD8^+^ T cells (51). Upregulation of these genes was confirmed by qPCR (Supplementary Fig. S6A), and ELISA demonstrated that secretion of CXCL9 and CXCL10 was strongly increased in cancer cells co-treated with TAK-243 and IFN-γ (Supplementary Fig. S6B). Importantly, surface expression of MHC-I in cancer cells was significantly increased after *Uba1* depletion or inhibition, both *in vitro* with IFN-γ stimulation (Fig. 5E; Supplementary Fig. S6C) and *in vivo* (Fig. 5F; Supplementary Fig. S6D–S6E). This supports the notion that UBA1 inhibition enhances antigen presentation and thus tumor recognition by CD8^+^ T cells, as surface MHC-I without loaded antigen is unstable at physiological temperatures (52). In agreement with this, a recent study incorporating three CRISPR screens identified UBA1 as one of the 44 candidate negative regulators of both surface MHC-I and surface MHC-I–peptide complex expression (53). Of note, as genes regulated by IFN-γ (54), MHC-II genes also showed increased expression upon UBA1 inactivation, both *in vivo* (Supplementary Fig. S5D) and *in vitro* (Supplementary Fig. S6F). This observation was consistent with the increased presence of CD4^+^ T cells in *Uba1*-depleted tumors (Supplementary Fig. S3D) and the involvement of CD4^+^ T cells in mediating the control of tumor growth in these *Uba1*-depleted tumors (Fig. 2I; Supplementary Fig. S2E).

The type-I IFN pathway also has the potential to elicit a strong antitumor response (55). As the pathway was also consistently enriched in our sequencing data (Fig. 5A–D; Supplementary Fig. S5A–S5C), we examined how cells respond to IFN-β treatment upon UBA1 inhibition or depletion. As hypothesized, we found that UBA1-inhibited cells exhibited an upregulated response to IFN-β manifested by elevated signaling (Supplementary Fig. S6G) and expression of *Cxcl10* and MHC-I genes (Supplementary Fig. S6H and S6I), which was also confirmed in the *Uba1*-depleted cells (Supplementary Fig. S6J).

As UBA1 is an E1 enzyme at the apex of the ubiquitin-mediated proteasomal degradation pathway, we speculated that UBA1 altered response to IFN via regulating the stabilities of effector proteins in key signaling pathways. We thus performed mass spectrometry analysis on cancer cells treated with TAK-243 to discover candidate targets and found that IFN pathway effectors, including JAK1 and IFNGR1, were stabilized by TAK-243 treatment (Fig. 5G). To identify candidates crucial for IFN response, we further performed CRISPR–Cas9 knockout screens targeting all the genes that were robustly upregulated by TAK-243 (Fig. 5G), using MHC-I expression as an indicator of IFN signaling activation. As expected, we found that the IFN pathway effectors, *Jak1* and *Ifngr1*, were MHC-I positive regulators in UBA1-inhibited and IFN-γ–stimulated cells, with *Jak1* being the top hit [Fig. 5H (left)]. In IFN-β–stimulated cells, *Jak1*, but not *Ifngr1*, was also one of the top MHC-I–positive regulators [Fig. 5H (right)], showing that JAK1 is crucial for both type-I and type-II IFN signaling (56), elevated by UBA1 inhibition. Time course immunoblot analysis validated that JAK1, a short-lived protein (57), was strongly stabilized by UBA1 inhibition with TAK-243 (Supplementary Fig. S6K). By contrast, JAK2 and STAT1 levels were not obviously changed, whereas IRF1 was susceptible to UBA1-independent degradation (Supplementary Fig. S6K). In response to IFN-γ, as expected, we observed an upregulation of JAK1 protein upon UBA1 inhibition or depletion, and p-STAT1 and IRF1 were strongly increased (Fig. 5I). Importantly, knockout of *Jak1* abolished the induction of surface MHC-I (Fig. 5J; Supplementary Fig. S6I) or chemokines (Supplementary Fig. S7A) in response to co-treatment with TAK-243 and IFN-γ (Fig. 5J) or with TAK-243 and IFN-β (Supplementary Fig. S6I). Additionally, *Jak1* loss significantly reversed tumor growth impairment mediated by the combination of TAK-243 and anti–PD-1 (Fig. 5K). We also examined the levels of JAK1 in *Uba1*-overexpressing cells, and as expected, overexpression of *Uba1* decreased JAK1 and reduced response to IFN-γ, as indicated by the reduction of p-STAT1 and IRF1 (Supplementary Fig. S7B). Importantly, in *Uba1*-overexpressing tumors, we detected diminished surface expression of tumor-specific MHC-I (Supplementary Fig. S7C) and reduction of mRNA levels of *Cxcl10*, *Cxcl9*, and MHC-I genes compared with the control (Supplementary Fig. S7D). The induction of response to IFN-γ or IFN-β by UBA1 inhibition was also observed in an independent murine model, demonstrating that the phenomenon was not model specific (Supplementary Fig. S7E and S7F). Collectively, these data are consistent with the notion that JAK1 is a key effector for both type-I and type-II IFN signaling (56), supporting that UBA1 modulates JAK1 stability leading to the observed alterations in IFN pathways in *Uba1*-modulated or *Uba1*-inhibited models (Fig. 5A–D; Supplementary Fig. S5A–S5C).

Of note, in mass spectrometry analysis, we identified previously reported (23) UBA1 targets, such as p53, MCL1, and c-Jun (Supplementary Fig. S7G). To exclude the possibility that JAK1 upregulation was due to stabilization of p53 or induction of apoptosis, we further performed a time course immunoblot analysis assessing the levels of JAK1, p53, and apoptosis (cleaved PARP) in cells treated with TAK-243 at different concentrations and for different durations (Supplementary Fig. S7H). We observed that under UBA1 inhibition, upregulation of JAK1 was stronger and happened earlier than upregulation of p53 or emergence of cleaved PARP (Supplementary Fig. S7H). Therefore, upregulation of JAK1 by UBA1 inhibition is unlikely to be a secondary effect caused by p53 upregulation or apoptosis. Intriguingly, MHC-I genes H2-K1 and H2-D1 were modestly stabilized by UBA1 inhibition (Supplementary Fig. S7I). However, an important component of surface MHC-I, B2M, was not significantly changed (Supplementary Fig. S7I). Importantly, in *Jak1*-null cells, TAK-243 treatment or co-treatment of TAK-243 and IFN-γ (Fig. 5J) failed to induce MHC-I surface expression, underscoring that JAK1-STAT1 signaling, but not the stabilization of H2-K1 and H2-D1, was crucial for surface MHC-I expression that was upregulated by UBA1 inhibition.

**Figure 6.**
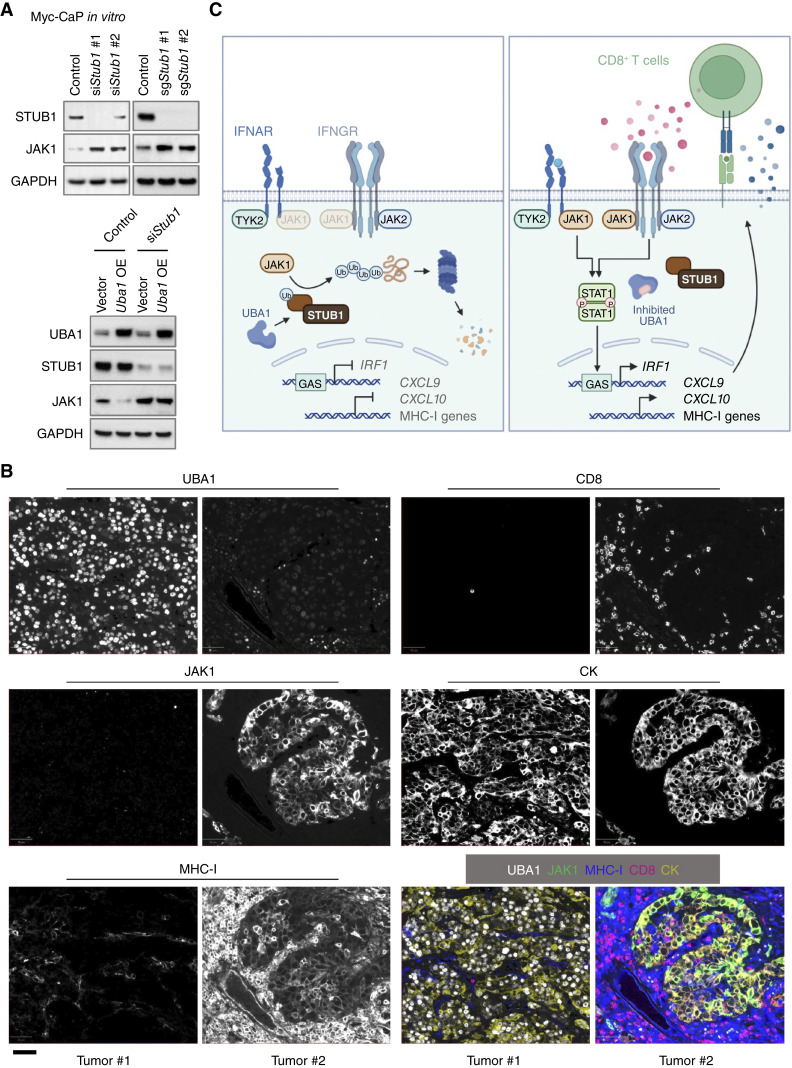
Depletion of *Stub1* upregulates JAK1. **A,** Top: immunoblot analysis assessing levels of the indicated proteins in Myc-CaP cells that received distinct siRNAs or sgRNAs targeting *Stub1*. Nontargeting siRNA or sgRNA was used as control, respectively. Bottom: immunoblot analysis assessing levels of the indicated proteins in the indicated Myc-CaP cells. OE, overexpression. **B,** Representative images of multiplex immunofluorescence staining for the indicated proteins in UBA1-high and UBA1-low human tumor samples. CK, pan-cytokeratin. Scale bar, 50 µm. **C,** Schematic showing that UBA1 upregulation in tumor cells facilitates STUB1-mediated proteasomal degradation of a key IFN sensor, JAK1, resulting in low expression of IFN-stimulated genes and thus an immune-cold tumor microenvironment (left). By contrast, inhibition of UBA1 elevates JAK1 and enhances response to IFNs, contributing to the formation of an immune-hot tumor microenvironment (right).

We also confirmed that UBA1-mediated JAK1 degradation was proteasome-dependent, as UBA1 inhibition failed to elevate JAK1 when the proteasome was inhibited with bortezomib (Supplementary Fig. S8A). Furthermore, inactivation of UBA1 strongly reduced ubiquitinated JAK1 both *in vitro* and *in vivo* (Supplementary Fig. S8B). STUB1 has been reported as an E3 ligase mediating JAK1 degradation (58) and implicated as an MHC-I negative regulator (53). Consistent with the literature, we found that knockdown or knockout of *Stub1* elevated JAK1 [Fig. 6A (top)] and MHC-I levels (Supplementary Fig. S8C). Importantly, depleting *Stub1* counteracted the decrease in JAK1 levels [Fig. 6A (bottom)] and increase in ubiquitinated JAK1 (Supplementary Fig. S8D) caused by *Uba1* overexpression. Collectively, the data support that STUB1 is an E3 ligase, responsible for UBA1-mediated JAK1 degradation.

We next evaluated the impact of *Uba1* depletion or inactivation on JAK1 levels *in vivo*. As expected, we found that tumors with *Uba1* depletion or inactivation displayed significant reduction of ubiquitinated proteins (Supplementary Fig. S9A). Importantly, *Uba1* depletion or inactivation strongly increased JAK1 and IRF1, a downstream effector of JAK1/STAT1 signaling, *in vivo* (Supplementary Fig. S9A). We also investigated the impact of TAK-243 administration on JAK1 and MHC-I expression in tumor-associated macrophages. Intriguingly, we found that UBA1 expression was markedly higher in tumor cells than in macrophages [Supplementary Fig. S9B (left)], accompanied by increased abundance of ubiquitinated proteins [Supplementary Fig. S9B (left)]. Although a single dose of TAK-243 administration markedly reduced the levels of ubiquitinated proteins in tumor cells, their levels in macrophages, which had inherently lower expression of UBA1 and ubiquitinated proteins, were less affected [Supplementary Fig. S9B (left)]. Consistently, JAK1 and MHC-I were markedly upregulated in tumor cells, but not in the macrophages, by TAK-243 administration (Supplementary Fig. S9B). Consistent with these findings, our assessment of clinical samples revealed that UBA1 expression was considerably lower in macrophages than in tumor cells (Supplementary Fig. S9C).

We next examined the tumor-specific expression of UBA1, JAK1, and MHC-I in clinical samples. We observed significantly reduced levels of JAK1 and MHC-I in tumors with high tumor-specific UBA1 expression compared with UBA1-low tumors (Supplementary Fig. S10A). Of note, virtually all high-UBA1–expressing tumors showed low expression of JAK1 [Supplementary Fig. S10A (right)]. We further subjected UBA1-high and UBA1-low human tumor samples to multiplex immunofluorescence staining (Fig. 6B). As expected, tumor cells with high UBA1 expression showed lower JAK1 and MHC-I expression (Fig. 6B). Moreover, although low-UBA1–expressing tumors were surrounded by and infiltrated with a high density of CD8^+^ T cells, tumors with high UBA1 levels were immune-cold with low abundance of CD8^+^ T cells (Fig. 6B). We next treated human cancer cells with TAK-243 or small interfering RNAs (siRNA) targeting *UBA1* and found that treatment with TAK-243 or knockdown of *UBA1* upregulated JAK1 levels; this was accompanied by an increased response to IFN-γ or IFN-β, manifested by upregulation of p-STAT1, IRF1, and surface MHC-I (Supplementary Fig. S10B–S10F). These data show that inhibition or depletion of *UBA1* also stabilizes JAK1 and enhances the IFN response in human cancer.

## Discussion

Using comprehensive bioinformatics analyses, we identified the expression of *UBA1*, a gene frequently gained in cancer, as being associated with low levels of intratumoral CD8^+^ T cells, ICB resistance, and poor survival in ICB-treated cohorts. Through the use of syngeneic murine cell lines with partial *Uba1* depletion and models of *Uba1* overexpression, we established that UBA1 facilitates tumor progression and diminishes intratumoral CD8^+^ T cell levels. Mechanistically, UBA1 facilitates STUB1-mediated proteasomal degradation of JAK1 in tumor cells. This process leads to reduced expression of IFN-stimulated genes and consequently contributes to an immune-cold tumor microenvironment (Fig. 6C).

Intriguingly, apart from the T cell chemokines CXCL9 and CXCL10, we observed upregulation of surface MHC-I expression upon UBA1 inhibition or depletion in various cancer models (Fig. 5E and F; Supplementary Figs. S7F, S10C and S10D). Surface MHC-I without antigen loaded is unstable at physiological temperatures (52); therefore, these data suggest that UBA1 inhibition increases antigen presentation and results in tumor recognition by CD8^+^ T cells. This is seemingly contradictory to an antigen-processing role for proteasomal protein degradation (59–61). However, accumulating evidence has demonstrated that a substantial number of antigens can be processed in a ubiquitin- or proteasome-independent manner, supporting a more sophisticated mechanism of generating MHC-I–peptide complexes than had been previously recognized (62–65). Thus, we speculate that although antigen processing may be partially compromised by UBA1 inhibition, a strong elevation of JAK1 and IFN signaling yields an overall outcome of increased MHC-I surface expression. In line with this, a recent study incorporating three CRISPR screens shows *UBA1* is one of the 44 candidates that negatively regulate expression of both surface MHC-I and surface MHC-I–peptide complexes (53).

In summary, our study (i) identifies UBA1 as a biomarker predictive of clinical outcomes in ICB cohorts; (ii) implicates UBA1 in mediating immune evasion, thus promoting tumor progression; (iii) defines JAK1 stabilization as a primary mechanism through which UBA1 inhibition influences immune responses; and (iv) nominates the UBA1–STUB1 axis as an immuno-oncology therapeutic target. Importantly, administration of the clinical UBA1 inhibitor TAK-243 enhances efficacy of ICB in a series of preclinical models.

Despite these impactful findings, our investigation on TAK-243 efficacy has been limited to preclinical settings. Two phase I clinical trials of single-agent TAK-243 were initiated: NCT03816319 is ongoing, and NCT02045095 was terminated due to realignment of sponsor priorities. NCT03816319 is assessing the recommended phase II dose and activity of TAK-243 in patients with relapsed or refractory acute myeloid leukemia, myelodysplastic syndrome, or chronic myelomonocytic leukemia. However, evaluation of co-inhibiting UBA1 and immune checkpoint has not been initiated clinically. Based on our findings, clinical trials should be undertaken to evaluate UBA1 inhibitors (such as TAK-243) in combination with ICB as a new strategy to boost immunotherapy response across multiple cancer types.

## Methods

### Cell Lines and Reagents

Cell lines were acquired as previously described (66, 67). B16-F10, Myc-CaP, TRAMP-C2, VCaP, and A375 were purchased from ATCC, and B16-BL6 was purchased from Riken. Cell lines were maintained following the instructions from the manufacturers. Pellets of all cell lines were regularly sent to Labcorp Cell Line Testing division (Burlington) for authentication. All cell lines were tested for *Mycoplasma* contamination every 2 weeks to assure that they remained *Mycoplasma* free. TAK-243 (formerly known as MLN7243) was purchased from MedChemExpress. Recombinant mouse IFN-γ (#485-MI), mouse IFN-β (#8234-MB), and human IFN-γ (#285-IF) were acquired from R&D Systems. *UBA1*-targeting siRNAs (catalog #4427038; assay ID s599 and s600), *Stub1*-targeting siRNAs (catalog #4390771; assay ID s80536 and s80537), and the non-targeting control (catalog #4390843) were acquired from Thermo Fisher Scientific.

### Stable Cell Lines with Gene Overexpression or Depletion

*Uba1* gene was amplified with cDNA derived from Myc-CaP cells and constructed into the backbone pLenti CMVie-IRES-BlastR (Addgene; #119863). Successful construction was confirmed by Sanger sequencing. The *Uba1*-carrying vector or empty vector (control) was then co-transfected with pRSV-REV (Addgene; #12253), pMDLg/pRRE (Addgene; #12251), and pMD2.G (Addgene; #12259) into HEK293T cells to generate virus. The virus media was filtered with a 0.22-µm filter to remove cell debris, then the target cells were infected with the virus in medium containing 4 μg/mL of polybrene (Sigma-Aldrich; #H9268). One day after infection, infected cells were selected with blasticidin S (Thermo Fisher Scientific; #A1113903) at 10 μg/mL. Cells with gene depletion were generated as described previously (68). Briefly, single-guide RNAs (sgRNA) targeting early exons of the target genes were checked for off-target prediction using Off-Spotter (https://cm.jefferson.edu/Off-Spotter/), and sgRNAs with weak off-target potential were selected. The target sequences of all sgRNAs in this study are provided in Supplementary Table S2. The lentiCRISPR v2 (Addgene; #52961) was used to carry the sgRNAs, and the Golden Gate reaction was used for vector construction. Sanger sequencing was performed to confirm successful insertion. The vector containing the sgRNA was next transfected into target cells using Lipofectamine 3000 (Thermo Fisher Scientific; #L3000001). One day after transfection, cells were selected with puromycin, and then single cells were plated into 96-well dishes with a cell sorter (Sony SH800S). Sublines derived from the single cells were next expanded. Depletion of the target gene was determined by Sanger sequencing and immunoblot. For generating GFP-labeled cancer cells, viral Lenti-GFP containing cytomegalovirus (CMV)-driven GFP was acquired from the Biomedical Research Core Facilities at the University of Michigan (U-M) and used to infect the target cells. Two days after infection, the GFP-expressing cells were sorted using a cell sorter. All stable cell lines were assessed for *Mycoplasma* contamination every 2 weeks to assure that they remained *Mycoplasma* free.

### Flow Cytometry Analysis

Measurement of MHC-I surface expression in cultured cancer cells was performed as described previously (68). In brief, the cells were trypsinized and resuspended in MACS buffer (PBS containing 2% FBS and 2 mmol/L EDTA), stained with a LIVE/DEAD stain, Zombie NIR (BioLegend; #423106), and fluorophore-conjugated anti–MHC-I antibody. The cells were washed with 2 mL MACS after staining and then fixed with 2% paraformaldehyde in PBS prior to the analysis on a flow cytometer (Sony SH800S). In this experiment, the following antibodies were used: anti–H-2Kd (BD Biosciences; #562004) and anti–H-2Dd (BD Biosciences; #553580) for CT26, and anti–H-2Kq (BD Biosciences; #742296) and anti–H-2Dq/H-2Lq (BD Biosciences; #744853) for Myc-CaP. For measuring human MHC-I, anti–HLA-A,B,C (clone w6/32; BioLegend; #311406) was used.

For staining of T cell intracellular markers, tissues were weighed and cut into small pieces. They were then placed in digestion buffer, PBS containing 2% FBS and 0.5 mg/mL collagenase D (Roche; #COLLD-RO) and 0.25 mg/mL DNase I (Roche; #10104159001) for digestion at 37°C for 30 minutes. They were then filtered with 70-μm cell strainers, and then the suspensions were laid onto a density gradient medium (Lymphoprep; StemCell Technologies; #07851) in centrifuge tubes. After centrifugation, cell layers at the interface were harvested and washed with MACS. The cells were next cultured in RPMI 1640 (Gibco; #11875093) containing 10% FBS, 50 U/mL penicillin–streptomycin, 10 mmol/L HEPES, 27.5 µmol/L β-mercaptoethanol, 200 ng/mL phorbol 12-myristate 13-acetate, 1,000 ng/mL ionomycin, 1× brefeldin A, and 1× monensin at 37°C for 4 hours. The cells were then washed one time with PBS, stained with Zombie Green (BioLegend; #423112) in PBS, blocked with anti-mouse CD16/32 (BioLegend; #156604) in MACS, and stained with surface antibodies in MACS for 12 minutes at room temperature. The cells were next washed one time with 2 mL MACS, then fixation and permeabilization were performed with the Foxp3/Transcription Factor Staining Buffer Set (Thermo Fisher Scientific; #00-5523-00) following the instructions from the manufacturer. The cells were next stained with intracellular markers for 12 minutes at room temperature, washed one time with 2 mL 1× permeabilization buffer, and analyzed on a flow cytometer (BD LSRFortessa cell analyzer) with Absolute Counting Beads (Thermo Fisher Scientific; #C36950) added for quantification. The following surface antibodies were used in this experiment: anti-CD45 (BD Biosciences; #550994), anti-CD3 (BioLegend; #100237), anti-CD90.1 (BD Biosciences; #563770), anti-CD90.2 (BioLegend; #140327), anti-CD8 (BioLegend; #100742), and anti-CD4 (BD Biosciences; #553051). The following intracellular antibodies were used in this experiment: anti-Ki67 (Thermo Fisher Scientific; #56-5698-82), anti-granzyme B (BioLegend; #372208), and anti–IFN-γ (BD Biosciences; #562333).

For quantifying memory CD8^+^ T cells, tissues were weighed, cut, digested, filtered, and laid onto the density gradient medium as described above. After centrifugation and harvesting the interface cells, they were washed one time with PBS, stained with Zombie NIR (BioLegend; #423106) in PBS, blocked with anti-mouse CD16/32 (BioLegend; #156604) in MACS, and stained with the following surface antibodies in MACS for 12 minutes at room temperature. The cells were washed one time with 2 mL MACS and analyzed on a flow cytometer (BD LSRFortessa cell analyzer) with Absolute Counting Beads (Thermo Fisher Scientific; #C36950) added for quantification. Antibodies used in this experiment included anti-CD8 (Thermo Fisher Scientific; #MA5-16759), anti-CD44 (BD Biosciences; #563736), anti-CD62L (BioLegend; #104418), and anti-KLRG1 (BioLegend; #138412).

For assessing CD8^+^ T cell depletion after treatment of anti-CD8α antibody, 50 to 100 μL of blood was collected from the tail in an EDTA-coated tube. Anti–mouse CD16/32 (BioLegend; catalog #156604) was added to the blood and then the following antibodies used: anti-CD45 (BD Biosciences; #550994), anti-CD3 (BioLegend; #100237), anti-CD90.1 (BD Biosciences; #563770), anti-CD8 (BioLegend; #100742), and anti-CD4 (BD Biosciences; #553051). Staining was performed for 12 minutes at room temperature, then RBC Lysis Buffer (BioLegend; #420301) was used to lyse the red blood cells. The samples were then washed with MACS, fixed with 2% paraformaldehyde in PBS, and measured on the BD LSRFortessa cell analyzer.

FlowJo v.10.8.1. was used to analyze all flow cytometry data.

### scRNA-seq

Tumor tissues were cut into small pieces and then digested with PBS containing 2% FBS and 0.5 mg/mL collagenase D (Roche; #COLLD-RO) and 0.25 mg/mL DNase I (Roche; #10104159001) at 37°C for 30 minutes. Tissues were then filtered with 70-μm cell strainers, and then the cells were washed one time with PBS. Next, the cells were stained with Zombie NIR (BioLegend; #423106) in PBS, blocked with anti–mouse CD16/32 (BioLegend; #156604) in MACS, and stained with anti-CD45 (BD Biosciences; #550994) in MACS for 12 minutes at room temperature. Live CD45^+^ cells were then sorted with a cell sorter (Sony SH800S). For pathway enrichment and identifying differently expressed genes (DEG) in malignant cells, live CD45^−^ cells were mixed 1:1 with live CD45^+^ cells, prior to subjection to scRNA-seq. Sequencing was performed using the Chromium Next GEM Single Cell 3′ HT Kit v3.1 (Dual Index), a product from 10× Genomics, following the instructions from the manufacturer. The sequencing was performed with the Illumina NovaSeq.

Per-sample FASTQ files were generated from the raw base call files using Cell Ranger (69) *mkfastq*. The raw gene count matrices were generated from the FASTQ files using the Cell Ranger count command and the 10× Genomics supplied *mm10* reference. *Seurat* (70) and *scDblFinder* (71) were used to remove cells that were either classified as doublets, had counts of less than 200 genes, or had more than 5% mitochondrial genes. The samples were then processed using Seurat methods, including log-normalization, scaling, PCA dimensionality reduction, Uniform Manifold Approximation and Projection dimensionality reduction, and clustering of cells. Malignant cell clusters were identified as clusters having a low expression of *Cd45* (also known as *Ptprc*) and high expression of *Ar* and *Myc*. Immune cell clusters were identified as clusters having high expression of *Cd45*. The immune cell clusters were further annotated using a previously published single-cell mice immune cell dataset (72) and *Seurat* label-transfer methods, *FindTransferAnchors* and *TransferData*. Although basophil clusters were not present in the reference dataset, they were identified in the samples as clusters with high expressions of *Mcpt8* and *Cd200r3*. The top 10 DEGs were identified using *FindAllMarkers*. Control and treatment samples were integrated using 2,000 integration features and *Seurat IntegrateData*. T-cell clusters were separated and reprocessed to identify more granular subclusters. The subclusters were annotated based on their top 10 DEGs, in combination with manually assessing the difference of *Cd8a*, *Cd8b*, *Rora*, and *Cd4* expression. Pseudobulk matrices for each sample were generated and used for pathway enrichment analysis between conditions. Gene set ranking [log_2_ fold change × −log_10_ (*P* value)] with the *fgsea* package was used for the gene set enrichment analysis. The hallmark pathway analysis was based on the Molecular Signatures Database (73, 74).

### Animal Experiments

The U-M Institutional Animal Care and Use Committee approved all experimental protocols. Subcutaneous tumor models were established by injecting B16-F10 cells (3 × 10^5^), B16-BL6 cells (3 × 10^5^), CT26 cells (5 × 10^5^), MC38 cells (3 × 10^6^), Myc-CaP cells (3 × 10^6^), or TRAMP-C2 cells (3 × 10^6^) in a volume of 50 µL to both flanks of their syngeneic immunocompetent mice or SCID mice. Female BALB/c, male (for TRAMP-C2) or female C57BL/6, male FVB, and SCID mice, aged 6 to 8 weeks, were used. All animals were acquired from The Jackson Laboratory. Cells were washed and resuspended in PBS prior to injection. Tumor volume was measured 5 to 9 days after tumor cell injection and conducted every 2 to 4 days, using calipers. The formula (*W*^2^ × *L*)/2, in which W is minor tumor axis and L the major, was used for tumor volume calculation. All mice were pathogen free and maintained in a cycle of 12-hour light/dark.

### *In Vivo* Treatments

Mice were randomized when tumors reached 35 to 100 mm^3^. Preparation and intravenous administration of TAK-243 followed the previously described protocol (23). Administration of TAK-243 was performed at 25 mg/kg, two times a week. Anti–PD-1 (clone RMP1-14) and its isotype control were from Bio X Cell and administered intraperitoneally, two times a week, at 50 μg per mouse for MC38 tumor models and at 200 μg per mouse for all the other models. For depletion of T cells, anti–mouse CD8α (clone 2.43), anti–mouse CD4 (clone GK1.5), or their isotype controls from Bio X Cell were administrated intraperitoneally on 1 day before tumor cell inoculation or co-treatment and TAK-243 and anti–PD-1, at 400 μg per mouse, and subsequently 100 μg per mouse one time every 3 days until the end of the experiment.

### Evaluation of *In Vivo* Drug Synergism

Evaluation of *in vivo* drug synergism was performed with a publicly available tool, CombPDX (https://licaih.shinyapps.io/CombPDX/), following the tutorial (44). Combination indexes were generated under the Highest Single Agent reference model. A combination index larger than zero was defined as supra-additive (synergistic; ref. 44).

### Cell Sorting

Tumor tissues were cut into small pieces and then digested with PBS containing 2% FBS and 0.5 mg/mL collagenase D (Roche; #COLLD-RO) and 0.25 mg/mL DNase I (Roche; #10104159001) at 37°C for 30 minutes. Tissues were then filtered with 70-μm cell strainers, then washed one time with PBS. Next, the cells were stained with Zombie NIR (BioLegend; #423106) in PBS, blocked with anti–mouse CD16/32 (BioLegend; #156604) in MACS, and stained with anti-CD45 (BD Biosciences; #550994) and anti-F4/80 (BD Biosciences; #565613) in MACS for 12 minutes at room temperature. The live CD45^+^ F4/80^+^ cells were then sorted using a cell sorter (SONY SH800S), and the live GFP-labeled tumor cells were sorted by GFP expression. The sorted cells were then subjected to further analysis.

### Human Studies

The U-M Institutional Review Board approved the acquisition and use of clinical data in this study. Patients were recruited at the U-M Hospital in Ann Arbor, Michigan. Patient samples were sequenced through the MI-ONCOSEQ clinical sequencing program (30, 75–77) at the Michigan Center for Translational Pathology. Data from pretreatment samples were analyzed for predicting treatment resistance and survival. Treatment response was assessed using RECIST1.140 criteria, with pseudo progression [imRECIST criteria (78)] excluded. The Michigan Center for Translational Pathology Clinical Laboratory Improvement Amendments–compliant laboratory performed sequencing of patient samples with approved protocols, in line with recognized ethical guidelines, as described previously (76, 77, 79). Written informed consents were obtained from the patients. Population characteristics of the cohort are shown in Supplementary Table S3.

### Bulk RNA-seq

After checking the quality of RNAs on an Agilent Bioanalyzer using the Eukaryote Total RNA Nano kit (Agilent Technologies; #5067-1511), the KAPA RNA HyperPrep Kit with RiboErase (Roche Sequencing Solutions; catalog #08098140702) was used to build the libraries with a total of 800 ng RNA for each sample, following the user’s manual. Briefly, enzymatic digestion was used to remove ribosomal RNAs, then the RNAs were fragmented with heat in fragmentation buffer. The 200- to 300-bp fragmented RNAs were next converted to cDNAs using reverse transcriptase and random primer. Second strands were next synthesized to obtain double-stranded cDNAs. New England Biolabs adapters were attached and then DNAs were amplified using the KAPA HiFi HotStart mix and New England Biolabs dual barcode. Library quality was assessed with Agilent Bioanalyzer using DNA 1000 Kit (Agilent Technologies; catalog #5067-1504), and NovaSeq 6000 was then used for sequencing. Data analysis was performed with packages *limma* (80, 81) and *edgeR* (82). Gene set ranking [log_2_ fold change × −log_10_ (*P* value)] with the *fgsea* package was used for gene set enrichment analysis. The hallmark pathway analysis was based on the Molecular Signatures Database (73, 74).

### Analysis of RNA-seq Data

Public bulk RNA-seq data from ICB-treated cohorts were downloaded from the cBioPortal (https://www.cbioportal.org/; refs. 83–85), the Tumor Immunotherapy Gene Expression Resource (http://tiger.canceromics.org/#/), the Kaplan–Meier plotter (https://kmplot.com/analysis/; ref. 86), or directly from the research article. Main datasets used in this study include two metastatic melanoma datasets [Snyder and colleagues (87) and Van Allen and colleagues (34)], a metastatic clear-cell renal cell carcinoma dataset [Miao and colleagues (36)], a glioblastoma dataset [Zhao and colleagues (35)], and a non–small cell lung cancer dataset [Jung and colleagues (37)]. Data from pretreatment samples were analyzed to determine if expression of *UBA1* was predictive of treatment response and patient survival. The best cutoff distinguishing high or low expression was used in the dichotomized analysis. The CTL signature (28) was composed of *CD8A*, *CD8B*, *GZMA*, *GZMB*, and *PRF1*, and the effector CD8 T-cell signature (32, 33) was composed of *CD8A*, *IFNG*, *GZMA*, *GZMB*, *TBX21*, *CXCL9*, *CXCL10*, and *PRF1*.

### qRT-PCR

QIAzol Lysis Reagent was used to lyse cells, and the RNeasy Mini Kit (QIAGEN) was used for RNA extraction, following the instructions from the manufacturer. The Maxima First Strand cDNA Synthesis Kit (Thermo Fisher Scientific; #K1671) was next used to obtain cDNAs, and the Fast SYBR Green Master Mix (Thermo Fisher Scientific; #4385612) was used for qPCR. qPCR was conducted in a 386-well format on QuantStudio 5 or 7 Pro system (Thermo Fisher Scientific). *ACTB* was used as a control for normalization, and 2^−ΔΔCT^ was used to determine the relative abundance of the target transcripts. Information for the primers used in this experiment are given in Supplementary Table S4.

### CRISPR/Cas9 Knockout Screens

The sgRNAs targeting genes upregulated by TAK-243 treatment were designed, synthesized, and constructed to pLentiGuide-Puro (GenScript), by GenScript. Six sgRNAs were designed for each gene. Myc-CaP cells were infected with virus carrying pLentiCas9-Blast (GenScript). After blasticidin (10 µg/mL) selection, expression of Cas9 was confirmed with immunoblotting. The Cas9-expressing cells were then infected with virus carrying the library at multiplicity of infection less than 0.3. Five days after puromycin (10 µg/mL) selection, the cells were treated with 75 nmol/L TAK-243 for 4 hours, and then 1 ng/mL of IFN-γ or 0.1 ng/mL of IFN-β for an additional 18 hours. After harvesting a pre-sort bulk population, the cells were stained with Zombie NIR (BioLegend; #423106), anti–H-2Kq (BD Biosciences; #742296), and anti–H-2Dq/H-2Lq (BD Biosciences; #744853). Cells with the highest 10% of MHC-I expression from the live cell population were sorted with a cell sorter (SONY SH800S). Sufficient cells were harvested to ensure 500× coverage. Genomic DNAs were extracted from the frozen pre-sorted and sorted cell pellets using the DNeasy Blood & Tissue Kit (QIAGEN, 69504), following manufacturer’s instructions. The sgRNAs were amplified with two rounds of PCR using Herculase II Fusion DNA Polymerase (Agilent Technologies, 600677) and the following primers:

First-round forward primer: TTTGCATATACGATACAAGGCTG;
First-round reverse primer: TCAAGATCTAGTTACGCCAAGC;
Second-round forward primer: TTTCTTGGGTAGTTTGCAGTTTT;
Second-round reverse primer: TCAAGATCTAGTTACGCCAAGC.

The PCR products were purified and size-selected using the Select-a-Size DNA Clean & Concentrator Kit (Zymo Research, D4080) and then Novex TBE Gels, 6%, 10-well (Thermo Fisher Scientific, catalog #EC6265BOX). The purified PCR products were then subjected to deep sequencing. Putative MHC-I regulators were identified by comparing sgRNA abundance among the top 10% of the cells with the highest MHC-I expression and pre-sorted populations. The sgRNAs with less than 50 reads in the pre-sorted populations were filtered, and genes with less than three targeting sgRNAs remaining were removed. Data from the CRISPR screen in Myc-CaP treated with TAK-243 + IFN-γ or TAK-243 + IFN-β are given in Supplementary Table S5 or Table S6, respectively.

### IHC

After paraffin embedding, tumor tissues were sectioned, deparaffinized, and rehydrated. Sodium citrate buffer (10 mmol/L sodium citrate, 0.05% Tween 20, pH 6.0) was then used for antigen retrieval. Sections were next treated with 3% H_2_O_2_ and blocked with 5% goat serum diluted in PBS. After blocking, samples were incubated with primary antibody overnight at 4°C, followed by washing with PBST buffer (0.1% Tween 20 in PBS). Sections were then stained with secondary antibody for 1 hour, washed with PBST, developed with 3,3′-diaminobenzidine (Vector Laboratories, SK-4100), and incubated with hematoxylin for 2 minutes for counterstaining. After mounting, images of sections were acquired with a microscope. Quantification was conducted after deconvoluting the layer of brown from the image in a downloadable online software: Fiji (ImageJ; https://imagej.net/software/fiji/downloads). Scoring for UBA1 levels was independently performed by two histologists. Primary antibodies used in this experiment included anti-UBA1 (Proteintech; #15912-1-AP), anti–HLA class 1 ABC (EMR8-5; ab70328), anti-JAK1 (Cell Signaling Technology; #3344), anti-IRF1 (Cell Signaling Technology; #8478), and anti-ubiquitinylated proteins antibody, clone FK2 (Sigma, 04-263). Secondary antibodies used in this experiment included horseradish peroxidase (HRP) goat anti–rabbit IgG secondary (Vector Laboratories; #MP-7451-15) and HRP goat anti–mouse IgG secondary (Vector Laboratories; #MP-7452-15).

### Immunoprecipitation

Immunoprecipitation was performed as described previously (88). Briefly, cells or minced tumor tissues were lysed with lysis buffer (50 mmol/L Tris-HCl pH 7.5, 120 mmol/L NaCl, 1 mmol/L EDTA, and 0.5% NP-40) supplemented with a protease inhibitor cocktail (Cell Signaling Technology; #5871). The lysates were then precleared with 0.25 µg of the control IgG (rat IgG2b isotype control; Thermo Fisher Scientific, 02-9288) and 20 μL Protein A/G PLUS-Agarose beads (Santa Cruz Biotechnology, sc-2003) at 4°C for 30 minutes. Cell lysates of 0.5 mg total proteins were incubated with 1.0 μg of anti-JAK1 antibody (R&D Systems, MAB4260) or the control IgG overnight with rotation at 4°C, and then 3-hour incubation with 20 μL Protein A/G PLUS-Agarose beads. The agarose beads were next washed five times with wash buffer (20 mmol/L Tris-HCl pH 7.5, 100 mmol/L NaCl, 1 mmol/L EDTA, 0.5% NP-40). The precipitated proteins were then denatured by the addition of NuPAGE LDS Sample Buffer (Thermo Fisher Scientific, NP0007) and heated at 70°C for 10 minutes. The proteins were then subjected to immunoblot analysis. The following antibodies were used in the immunoblot analysis: anti-GAPDH (Cell Signaling Technology; #3683S), anti-JAK1 (Cell Signaling Technology; #3332), and anti-ubiquitin (Cell Signaling Technology; #3936S).

### Immunofluorescence

Staining of CD8, UBA1, and DAPI was performed on the Ventana Discovery Ultra, and images were acquired using a florescence microscope, with pseudo-color: green for CD8, purple for UBA1, and blue for DAPI. Antibodies used in this experiment included anti-UBA1 (Cell Signaling Technology; #4890) and anti-CD8 (Roche; #790-4460). Staining of MHC-I was performed according to the previously described protocol (68). After paraffin embedding, tumor tissues were sectioned, deparaffinized, and rehydrated. Sodium citrate buffer (10 mmol/L sodium citrate, 0.05% Tween 20, pH 6.0) was then used for antigen retrieval. The sections were then blocked with goat serum (diluted to 5% in PBS) and incubated overnight at 4°C with the MHC-I antibody (Novus Biologicals, NB100-64952). After three washes with PBS, the sections were incubated with Alexa Fluor 488 goat anti–rat IgG (Jackson ImmunoResearch, 112-545-167), for 1 hour at room temperature. After mounting, images of the sections were acquired with a microscope. Quantification was performed using Fiji (ImageJ) software. Multiplex immunofluorescence was performed with the Lunaphore COMET system, using the following antibodies: anti-UBA1 (Cell Signaling Technology; #4890), anti-HLA Class 1 ABC (EMR8-5; ab70328), anti-JAK1 (Cell Signaling Technology; #3344), and anti-CD8, anti–pan-cytokeratin, and anti-CD68 antibodies were provided by Lunaphore.

### Immunoblot

Cells were washed with PBS and then lysed with RIPA buffer (Thermo Fisher Scientific; #89901) containing a protease inhibitor cocktail (Cell Signaling Technology; #5871). After sonication and removal of debris by centrifugation, protein concentration was determined using the Pierce BCA Protein Assay Kit (Thermo Fisher Scientific; #23227). The samples were then run on SDS-PAGE, followed by transferring to polyvinylidene difluoride membrane (Merck; #IPVH00010). Blocking was next applied to the membrane followed by incubation of the primary antibody at 4°C overnight. The membrane was then washed with TBST (0.1% Tween 20 in TBS) and incubated with HRP-linked secondary antibody. After the second wash, target proteins were visualized using the chemiluminescent substrate (Thermo Fisher Scientific; #34096) on the ChemiDoc XRS+ Imaging System (Bio-Rad). Antibodies used in this study included anti-GAPDH (Cell Signaling Technology; #3683S), anti-vinculin (Cell Signaling Technology; #18799S), anti-JAK1 (Cell Signaling Technology; #3344), anti-JAK1 (Cell Signaling Technology; #3332), anti-UBA1 (Cell Signaling Technology; #4891), anti-STAT1 (Tyr701; Cell Signaling Technology; #9167), anti-IRF1 (Cell Signaling Technology; #8478), and anti-ubiquitin (Abcam, ab134953).

### Mass Spectrometry

Cells were washed with PBS and then lysed with RIPA buffer (Thermo Fisher Scientific; #89901) containing a protease inhibitor cocktail (Cell Signaling Technology; #5871). After sonication and removal of debris by centrifugation, the protein concentration was determined using the Pierce BCA Protein Assay Kit (Thermo Fisher Scientific; #23227). A total of 75 mg of protein at 2 mg/µL for each sample was sent for mass spectrometry. The U-M Biomedical Research Core Facilities performed mass spectrometry and analyzed the data. Data from the mass spectrometry are given in Supplementary Table S7.

### Analysis of a Public scRNA-seq Dataset

A recently published scRNA-seq dataset from an ICB-treated melanoma cohort (89) was used in this study. The data were acquired from the KU Leuven Research Data Repository in the form of RDS files with metadata included. Cell-type annotations followed the original study. Malignant cells in pretreatment samples were used in the analysis. The Seurat package (v4.1.1) was used for single-cell analysis. Log1p was used for data normalization, and Seurat’s unsupervised graph-based clustering approach was used for clustering. FetchData function from Seurat was used to extract *UBA1* expression from each cell.

### Cell Proliferation Assay

*In vitro* proliferation of cancer cells was determined by the confluence of cells on 96-well dishes. The IncuCyte ZOOM system was used to capture cell images and calculate the confluence.

### Statistical Analysis

All data points were derived from distinct samples. Prism version 8 (GraphPad Software) was used for data analysis. Means ± SD or ± SEM were used for data presentation, as stated in the figure legends. A *P* value less than 0.05 was used to indicate statistical significance. All statistics were adjusted with Bonferroni correction.

### Data Availability

Sequencing data have been deposited to the NCBI Gene Expression Omnibus (accession numbers GSE253880 and GSE253810). All other data are given in the article or the Supplementary Materials.

## Supplementary Material

Supplementary Table S1 Supplementary Table S1. UBA1 protein expression and levels of intratumoral CD8+ T cells in tumor samples.

Supplementary Table S2 Supplementary Table S2. Target sequences of the sgRNAs used in this study.

Supplementary Table S3 Supplementary Table S3. Population characteristics of the MI-ONCOSEQ ICB cohort.

Supplementary Table S4 Supplementary Table S4. Sequences of primers used in RT-qPCR in this study.

Supplementary Table S5 Supplementary Table S5. Data from the CRISPR screen in Myc-CaP cells treated with TAK-243 + IFN-γ.

Supplementary Table S6 Supplementary Table S6. Data from the CRISPR screen in Myc-CaP cells treated with TAK-243 + IFN-β.

Supplementary Table S7 Supplementary Table S7. Data from the mass spectrometry in Myc-CaP cells treated with TAK-243.

Supplementary Figure S1 Supplementary Figure S1. High expression of UBA1 is associated with low levels of intratumoral CD8+ T cells and predictive of ICB resistance and poor survival in ICB cohorts.

Supplementary Figure S2 Supplementary Figure S2. UBA1 promotes tumor growth by mediating immune escape.

Supplementary Figure S3 Supplementary Figure S3. UBA1 diminishes intratumoral functional T cells.

Supplementary Figure S4 Supplementary Figure S4. UBA1 inhibition synergizes with anti-PD-1 therapy to control tumor growth.

Supplementary Figure S5 Supplementary Figure S5. UBA1 inactivation upregulates interferon signaling.

Supplementary Figure S6 Supplementary Figure S6. UBA1 inactivation upregulates interferon signaling via stabilizing JAK1.

Supplementary Figure S7 Supplementary Figure S7. UBA1 inactivation upregulates interferon signaling via stabilizing JAK1.

Supplementary Figure S8 Supplementary Figure S8. UBA1 mediates JAK1 ubiquitination via STUB1.

Supplementary Figure S9 Supplementary Figure S9. UBA1 inactivation upregulates interferon signaling *in vivo*.

Supplementary Figure S10 Supplementary Figure S10. UBA1 inactivation upregulates interferon signaling in human cancer.
