# Supplementary material for: The UBA1–STUB1 Axis Mediates Cancer Immune Escape and Resistance to Checkpoint Blockade
Source: Cancer Discov. 2024 Nov 14;15(2):363–81. doi: 10.1158/2159-8290.CD-24-0435 (PMC11803397; doi:10.1158/2159-8290.CD-24-0435)
Supplement: Supplementary Figure S1 — High expression of UBA1 is associated with low levels of intratumoral CD8+ T cells and predictive of ICB resistance and poor survival in ICB cohorts. [file cd-24-0435_supplementary_figure_s1_suppsf1.pdf]

Supplementary Figure S1

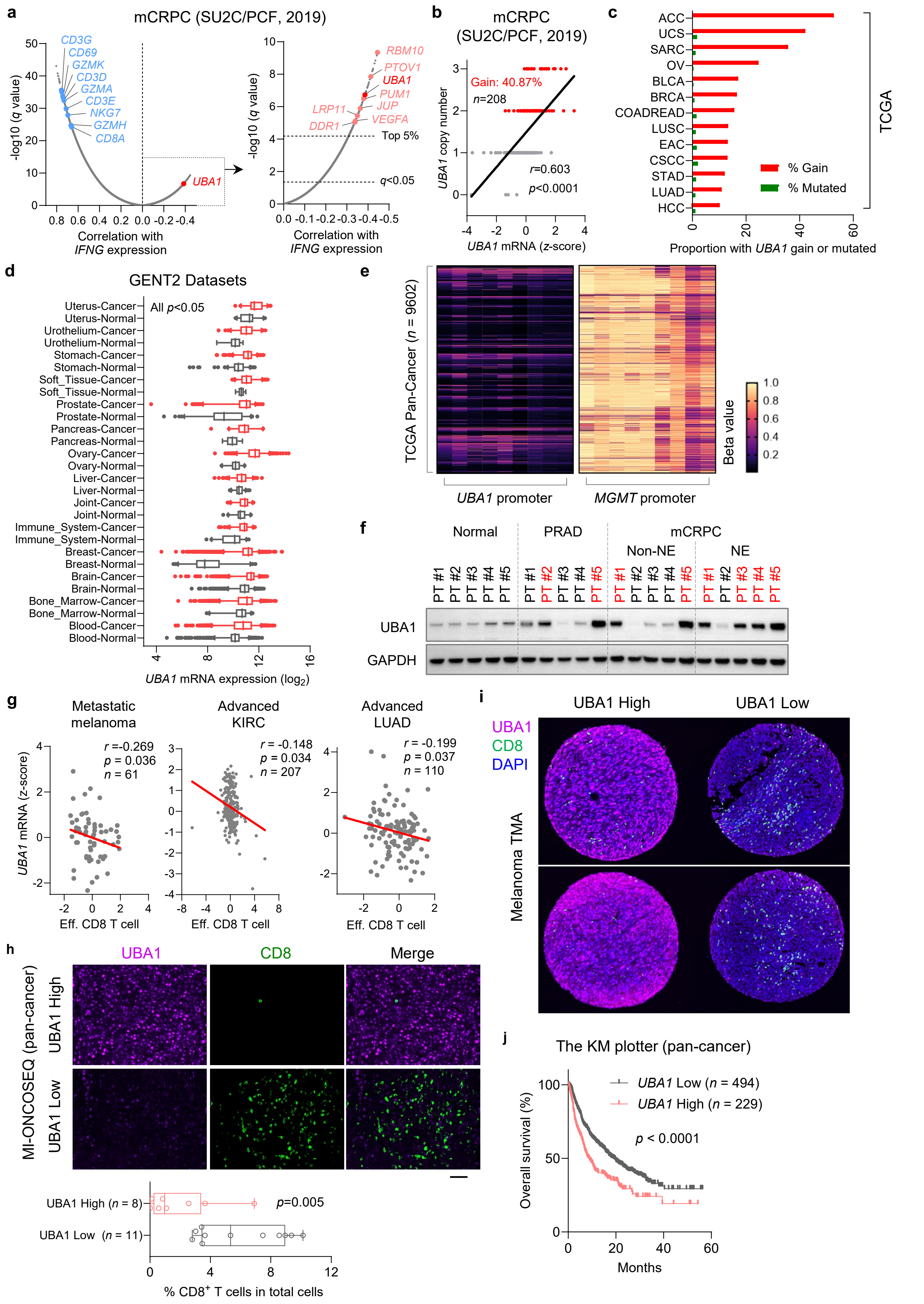

Supplementary Figure S1: **a**, Left: Spearman's correlation between mRNA expression of *IFNG* and other transcripts in the indicated metastatic castration-resistant prostate cancer (mCRPC) cohort. Genes that are associated with functional $CD8^+$ T cells are highlighted in blue. Right: Genes of which mRNA expression was inversely correlated with *IFNG* mRNA levels are shown. Genes that have been reported to be inversely associated with intratumoral amounts of $CD8^+$ T cells are highlighted in pink. **b**, Spearman's correlation between *UBA1* copy number and mRNA expression in the indicated mCRPC cohort. Frequency of copy-number-gain (gain) is shown. **c,** Proportion of cases with *UBA1* gains or mutations in the specified cancer types. Data were acquired from The Cancer Genome Atlas (TCGA). ACC: adrenocortical carcinoma; UCS: uterine carcinosarcoma; SARC: sarcoma; OV: ovarian serous cystadenocarcinoma; BLCA: bladder urothelial carcinoma; BRCA: breast invasive carcinoma; COADREAD: colorectal adenocarcinoma; LUSC: lung squamous cell carcinoma; EAC: esophageal adenocarcinoma; CSCC: cervical squamous cell carcinoma; STAD: stomach adenocarcinoma; LUAD: lung adenocarcinoma; HCC: hepatocellular carcinoma. **d**, *UBA1* mRNA expression in normal or cancer tissues in the indicated cancer types. Data are collected from the GENT2 database. GENT2: Gene Expression patterns across Normal and Tumor tissues. **e**, Methylation status on the promoters of the indicated genes. Data are acquired from UCSC Xena (https://xenabrowser.net/). **f**, Immunoblot analysis assessing UBA1 and GAPDH protein levels in normal, primary, or metastatic prostate cancer tissues. PRAD: prostate adenocarcinoma; NE: neuroendocrine. **g**, Spearman's correlation between mRNA expression of *UBA1* and the indicated signature in the indicated datasets. The metastatic melanoma dataset is a combination of two studies (Snyder et al. 2014 [87] and Van Allen et al. 2015 [34]). Data for advanced (stage III and IV) KIRC and LUAD were acquired from TCGA. KIRC: kidney renal clear cell carcinoma; LUAD: lung adenocarcinoma; Eff.: effector. **h**, Representative images (top) or quantification (bottom) of immunofluorescence assessing $CD8^+$ T cells in tumors with low or high UBA1 protein levels in the indicated cohort. Scale bar: 50 μm. **i**, Images of immunofluorescence for representative low or high UBA1-expressing tumors showing high or low amounts of intratumoral $CD8^+$ T cells, respectively, in a melanoma TMA. **j**, Overall survival of patients with tumors showing high or low pretreatment *UBA1* mRNA levels in the indicated cohort. Statistics were acquired by t-test in **d** and **h**, and log-rank test in **j**.
