# Supplementary material for: The UBA1–STUB1 Axis Mediates Cancer Immune Escape and Resistance to Checkpoint Blockade
Source: Cancer Discov. 2024 Nov 14;15(2):363–81. doi: 10.1158/2159-8290.CD-24-0435 (PMC11803397; doi:10.1158/2159-8290.CD-24-0435)
Supplement: Supplementary Figure S2 — UBA1 promotes tumor growth by mediating immune escape. [file cd-24-0435_supplementary_figure_s2_suppsf2.pdf]

Supplementary Figure S2

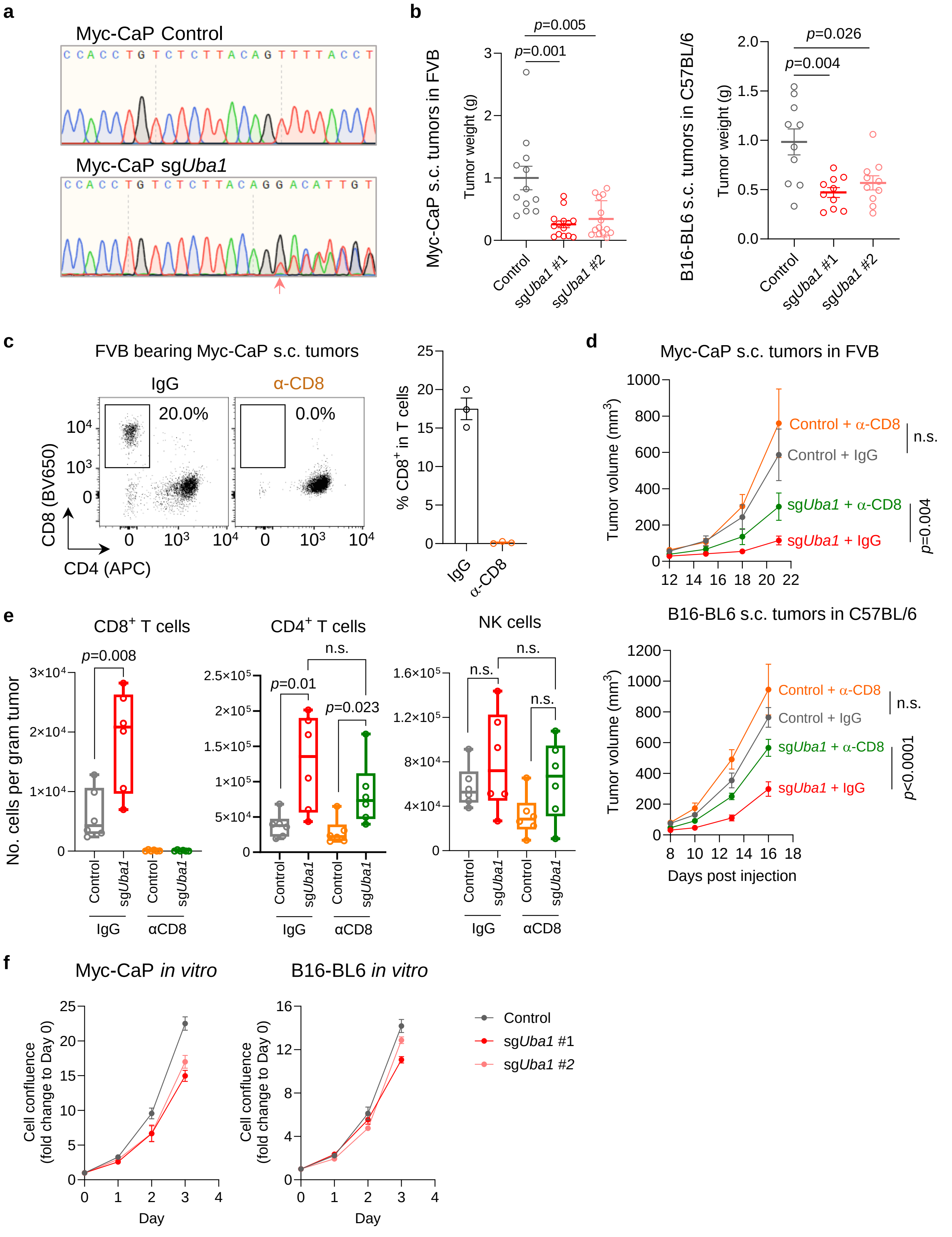

Supplementary Figure S2: a, Sanger sequencing on the region targeted by a single-guide RNA depleting *Uba1* (sg*Uba1*) in a representative clone derived from Myc-CaP cells. Control: a clone received a non-targeting single-guide RNA (control). An arrow highlighting the position that starts to show mixture of wild-type and mutated alleles is shown. **b**, Weights of subcutaneous (s.c.) tumors derived from Myc-CaP (left) or B16-BL6 (right) cells transfected with non-targeting single-guide RNA (control) or independent single-guide RNAs depleting *Uba1* (sg*Uba1* #1 and sg*Uba1* #2), in the indicated mice (*n* = 5–7 mice, per group). **c**, Quantification of flow cytometry measuring $CD8^+$ T cells in blood harvested from the indicated tumor-bearing FVB mice, four days post isotype control (IgG) or anti-mouse CD8 antibody (α-CD8) injection. **d**, Volumes of s.c. tumors established with injection of control or *Uba1*-depleted Myc-CaP (top) or B16-BL6 (bottom) cells to the indicated mice, with or without depletion of $CD8^+$ cells as in **c** (*n* = 5, per group). Data are representative of two distinct single-guide RNAs. **e**, Flow cytometry measuring the absolute numbers of $CD8^+$ T cells, $CD4^+$ T cells, and NK cells in the indicated tumors from mice with (α-CD8) or without (IgG) CD8 T cell depletion (*n* = 6 mice, per group). **f**, *In vitro* proliferation of Myc-CaP (left) or B16-BL6 (right) cells with or without depletion of *Uba1*. Data were acquired with technical triplicates.

Data are presented as mean ± SEM. Statistics were acquired by two-tailed Student's t test in b and e (n.s.: not significant) or by two-way ANOVA in d. Data in b are representative of two independent experiments.
