# Supplementary material for: The UBA1–STUB1 Axis Mediates Cancer Immune Escape and Resistance to Checkpoint Blockade
Source: Cancer Discov. 2024 Nov 14;15(2):363–81. doi: 10.1158/2159-8290.CD-24-0435 (PMC11803397; doi:10.1158/2159-8290.CD-24-0435)
Supplement: Supplementary Figure S3 — UBA1 diminishes intratumoral functional T cells. [file cd-24-0435_supplementary_figure_s3_suppsf3.pdf]

Supplementary Figure S3

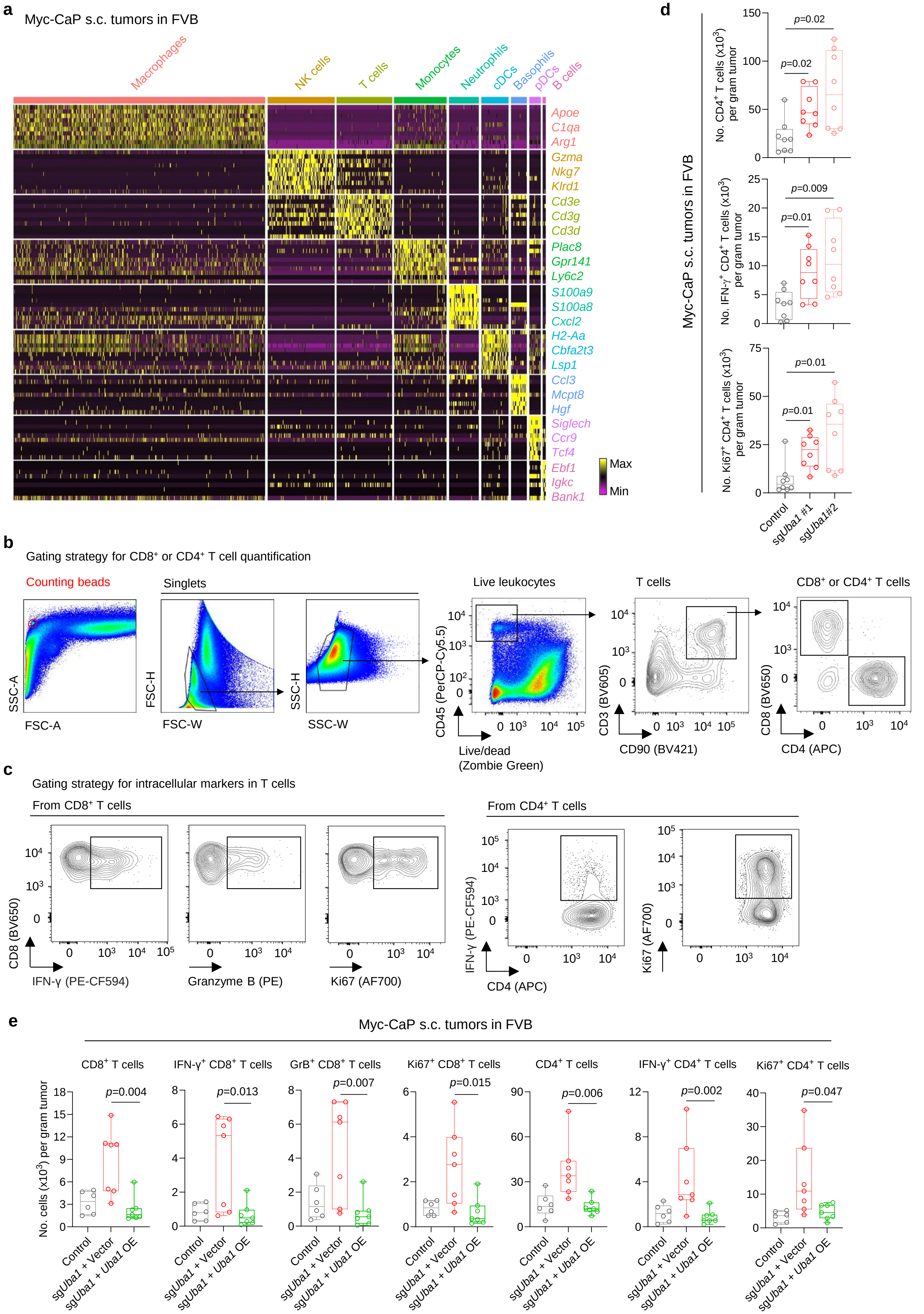

Supplementary Figure S3: a, Heatmap showing top 10 differentially expressed genes in each of the indicated clusters among $CD45^+$ immune cells from single cell RNA-sequencing. Three representative genes are shown on the right. **b–c**, Gating strategy for $CD8^+$ or $CD4^+$ T cell quantification (**b**) or for intracellular markers in T cells (**c**) in flow cytometry. **d**, Flow cytometry measuring the absolute numbers of $CD4^+$ T cells, IFN-γ^+ $CD4^+$ T cells, or $Ki67^+$ $CD4^+$ T cells in the indicated tumors. **e**, Flow cytometry measuring the absolute numbers of the indicated immune cells in the indicated tumors.

All data are presented as box and whisker plots. Statistics were acquired by two-tailed Student's t test. Data in **d** are pooled from two independent experiments.
