# Supplementary material for: The UBA1–STUB1 Axis Mediates Cancer Immune Escape and Resistance to Checkpoint Blockade
Source: Cancer Discov. 2024 Nov 14;15(2):363–81. doi: 10.1158/2159-8290.CD-24-0435 (PMC11803397; doi:10.1158/2159-8290.CD-24-0435)
Supplement: Supplementary Figure S4 — UBA1 inhibition synergizes with anti-PD-1 therapy to control tumor growth. [file cd-24-0435_supplementary_figure_s4_suppsf4.pdf]

Supplementary Figure S4

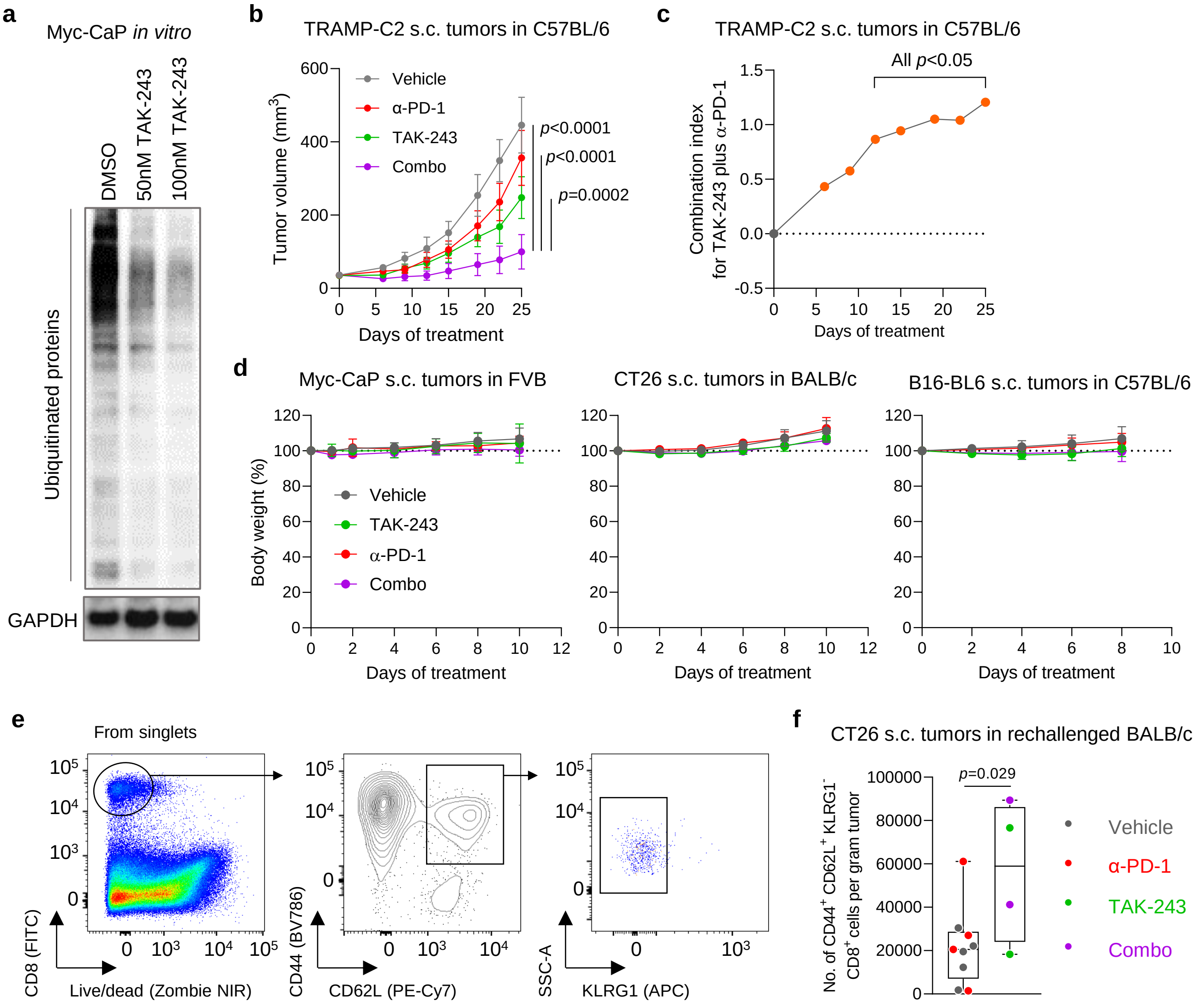

Supplementary Figure S4: a, Immunoblot analysis assessing levels of the indicated proteins in Myc-CaP cells treated with DMSO or TAK-243 at the indicated concentrations for 18 hours. **b**, Change of volume over time of the indicted tumor model, in the indicted mice treated with the indicated agents (*n* = 8 mice per group). **c**, Evaluation of drug synergism, using CombPDX [44], for the combination of TAK-243 and anti-PD-1 in the indicated model, treated as in **b**. **d**, Body weight of syngeneic mice bearing Myc-CaP (left), CT26 (middle), or B16-BL6 (right) subcutaneous (s.c.) tumors and treated with the indicated agents (*n* = 5–10 mice per group). TAK-243 was administered via intravenous (i.v.) injection. **e**, Gating strategy for memory $CD8^+$ T cells. **f**, Flow cytometry measuring the absolute numbers of memory $CD8^+$ T cells in CT26 s.c. tumors at rechallenged stage, in mice treated with the indicated agents. Combo: combined treatment of TAK-243 and anti-PD-1 (*n* = 4–9 mice, per group).

Data are presented as mean ± SEM in **b**, mean in **c**, mean ± SD in **d** and box and whisker plots in **f**. Statistics were acquired by two-way ANOVA in **b** or by two-tailed Student's t test in **f**.
