# Supplementary material for: The UBA1–STUB1 Axis Mediates Cancer Immune Escape and Resistance to Checkpoint Blockade
Source: Cancer Discov. 2024 Nov 14;15(2):363–81. doi: 10.1158/2159-8290.CD-24-0435 (PMC11803397; doi:10.1158/2159-8290.CD-24-0435)
Supplement: Supplementary Figure S5 — UBA1 inactivation upregulates interferon signaling. [file cd-24-0435_supplementary_figure_s5_suppsf5.pdf]

Supplementary Figure S5

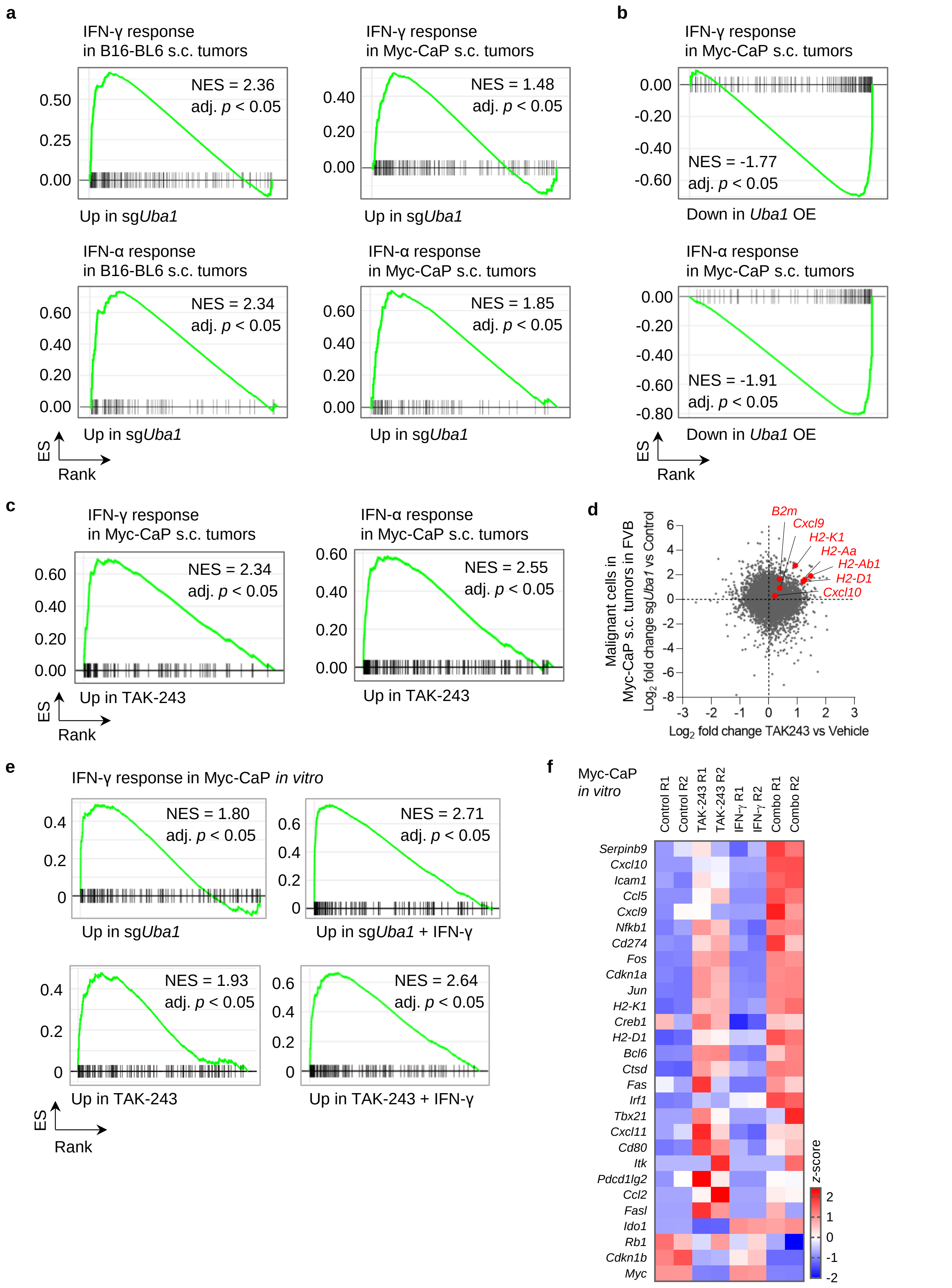

Supplementary Figure S5: a–c, Enrichment plots, from bulk RNA-sequencing, of the IFN-γ or IFN-α response pathways of the indicated tumors with *Uba1* depletion (sg*Uba1*) versus (vs.) control (**a**), with *Uba1* overexpression (OE) vs. control (empty vector; **b**), or from mice treated with TAK-243 or vehicle (**c**). **d**, Differentially expressed genes identified in the scRNA-seq in **Fig. 5c**. Representative genes that are responsive to IFN-γ are highlighted in red. TAK-243 was administered via i.v. injection, in **c–d**. **e**, Plots of the IFN-γ response pathway enriched by bulk RNA-sequencing of Myc-CaP cells with *Uba1* depletion (sg*Uba1*) vs. control (top), or Myc-CaP cells treated with or without 50 nM TAK-243 for 18 hours (bottom), and stimulated with or without IFN-γ. **f**, Heatmap showing mRNA levels of the indicated IFN-γ-regulated genes in Myc-CaP cells treated with or without 50 nM TAK-243 for 18 hours, and stimulated with or without IFN-γ. Combo: combination of TAK-243 and IFN-γ.
