# Supplementary material for: The UBA1–STUB1 Axis Mediates Cancer Immune Escape and Resistance to Checkpoint Blockade
Source: Cancer Discov. 2024 Nov 14;15(2):363–81. doi: 10.1158/2159-8290.CD-24-0435 (PMC11803397; doi:10.1158/2159-8290.CD-24-0435)
Supplement: Supplementary Figure S6 — UBA1 inactivation upregulates interferon signaling via stabilizing JAK1. [file cd-24-0435_supplementary_figure_s6_suppsf6.pdf]

Supplementary Figure S6

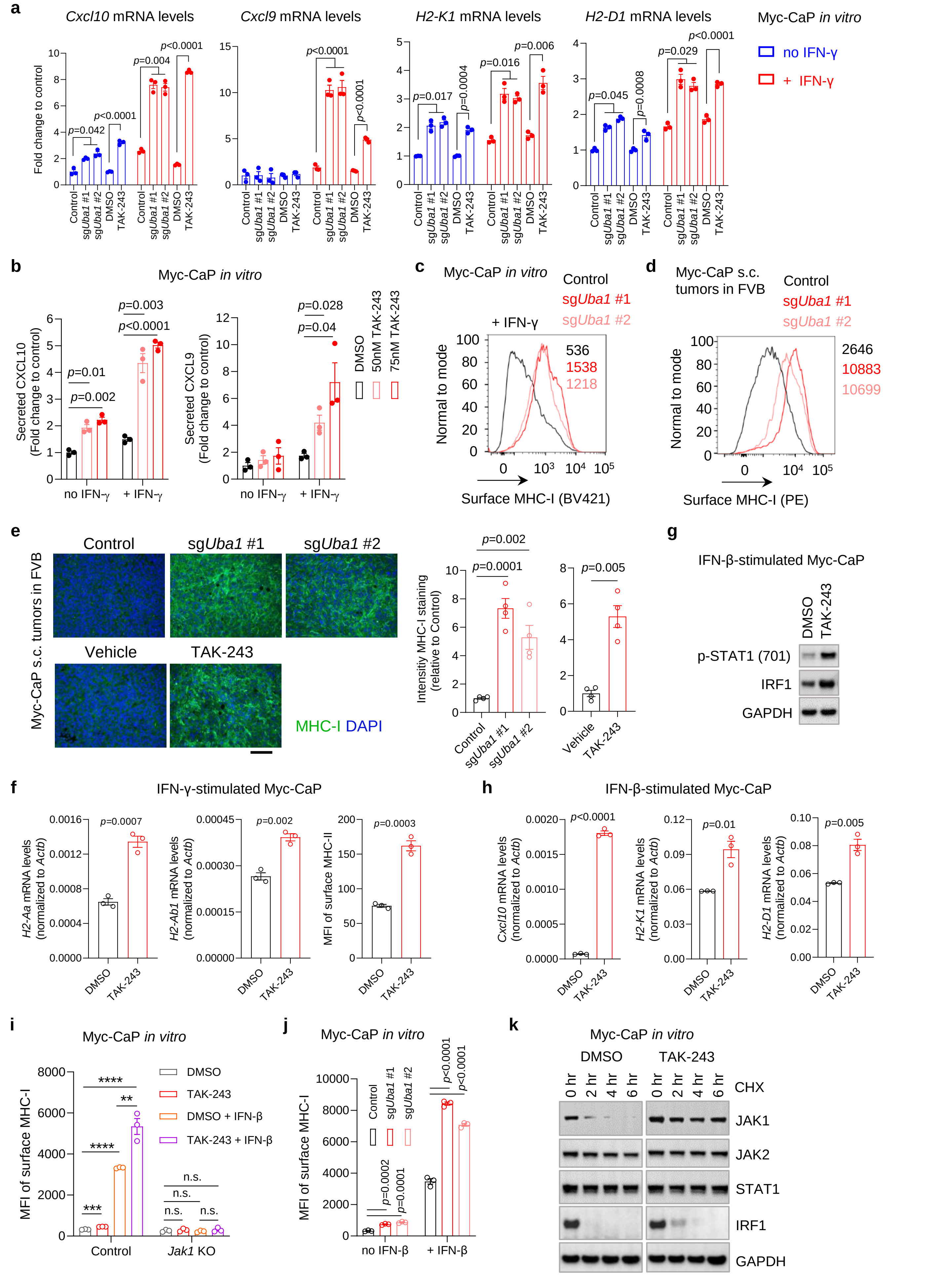

Supplementary Figure S6: a, Quantitative PCR measuring the mRNA levels of the indicated genes in Myc-CaP cells with *Uba1* depletion (sg*Uba1*) or UBA1 inhibition by 18 hours of 50 nM TAK-243 treatment, in the presence (red) or absence (blue) of IFN-γ stimulation. Non-targeting single-guide RNA or DMSO were used as controls, respectively. Data were acquired from technical triplicates, representative of two independent experiments. **b**, ELISA measuring secreted CXCL10 (left) and CXCL9 (right) from Myc-CaP cells treated with TAK-243 at the indicated concentration for 18 hours. Data were acquired from technical triplicates, representative of two independent experiments. **c**, Representative images of flow cytometry measuring surface expression of MHC-I in Myc-CaP cells that received independent single-guide RNAs depleting *Uba1* (sg*Uba1* #1 and sg*Uba1* #2). Cells were stimulated with 1 ng/mL IFN-γ for 18 hours. Cells receiving non-targeting single-guide RNA were used as control. Data are representative of three independent experiments. **d**, Representative images of flow cytometry measuring surface expression of MHC-I in GFP-labeled Myc-CaP tumor cells that were *Uba1*-depleted (sg*Uba1* #1 and sg*Uba1* #2) or control (n = 4 mice, per group). **e**, Representative images and quantification of immunofluorescence assessing MHC-I expression in the indicted tumors. **f**, Quantitative PCR measuring the mRNA levels of the indicated genes (left and middle) and flow cytometry measuring the surface expression of MHC-II (right) in Myc-CaP cells treated with 100 nM TAK-243 for 18 hours in the presence of IFN-γ stimulation. Data were acquired from technical triplicates, representative of two independent experiments. **g–h**, Immunoblot analysis assessing levels of the indicated proteins (**g**) or quantitative PCR measuring the mRNA levels of the indicated genes (**h**) in IFN-β-stimulated Myc-CaP cells treated with or without 100 nM TAK-243 for 18 hours. **i**, Surface expression of MHC-I measured by flow cytometry in the indicated cells treated with or without 50 nM TAK-243 and stimulated with or without IFN-β. **j**, Surface expression of MHC-I measured by flow cytometry in the indicated cells, stimulated with or without IFN-β. **k**, Immunoblot analysis assessing levels of the indicated proteins in Myc-CaP cells treated with 100 nM TAK-243 for four hours and subsequently 50 µg/ml of cycloheximide (CHX) for the indicated duration.

IFN stimulation was performed for 18 hours, with IFN-γ at 1 ng/mL and IFN-β at 0.1 ng/mL. Data are presented as mean ± SEM. Statistics were acquired by two-tailed Student's t test, except **a** (sg*Uba1* vs. control; by two-way ANOVA). **p<0.01; ***p<0.001; ***p<0.0001; n.s.: not significant.
