# Supplementary material for: The UBA1–STUB1 Axis Mediates Cancer Immune Escape and Resistance to Checkpoint Blockade
Source: Cancer Discov. 2024 Nov 14;15(2):363–81. doi: 10.1158/2159-8290.CD-24-0435 (PMC11803397; doi:10.1158/2159-8290.CD-24-0435)
Supplement: Supplementary Figure S7 — UBA1 inactivation upregulates interferon signaling via stabilizing JAK1. [file cd-24-0435_supplementary_figure_s7_suppsf7.pdf]

Supplementary Figure S7

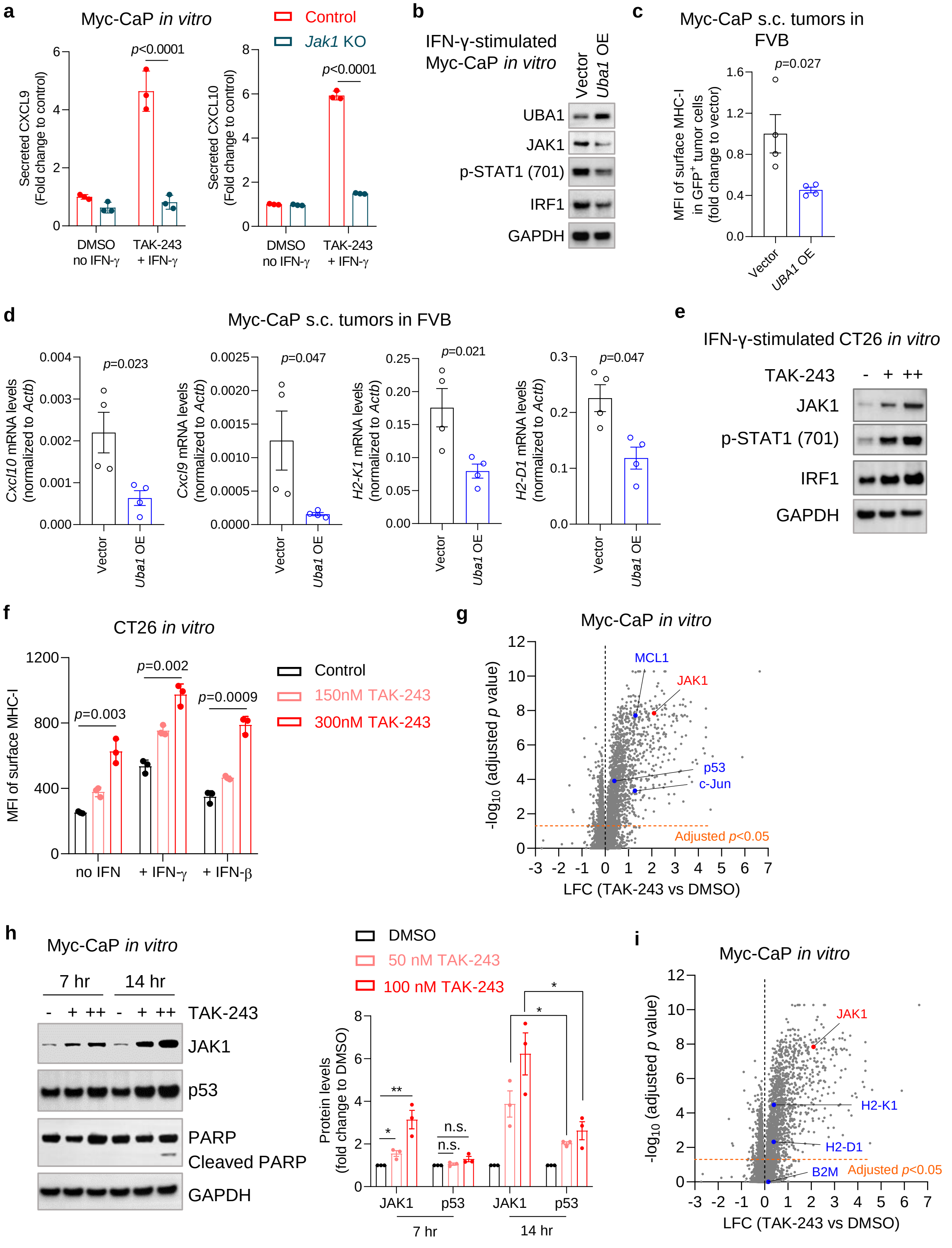

Supplementary Figure S7: **a**, ELISA measuring secreted CXCL9 (left) and CXCL10 (right) in Myc-CaP cells that received knockout of *Jak1* (*Jak1* KO) and were treated with 50 nM TAK-243 and stimulated with IFN-γ for 18 hours. Cells that received non-targeting single-guide RNA were used as control. Data are representative of two independent experiments with two distinct single-guide RNAs. **b**, Immunoblot analysis assessing levels of the indicated proteins in Myc-CaP cells infected with virus carrying *Uba1* overexpression (OE) or empty vector, in the presence of IFN-γ stimulation. **c,** Quantification of flow cytometry measuring surface expression of MHC-I in GFP-labeled Myc-CaP tumor cells that carried *Uba1* overexpression (OE) or empty vector (*n* = 4 mice, per group). **d**, Quantitative PCR measuring the mRNA levels of the indicated genes in tumors derived from Myc-CaP cells with *Uba1* overexpression (OE) or empty vector (*n* = 4 mice, per group). **e**, Immunoblot analysis assessing levels of the indicated proteins in CT26 cells treated with 150 nM or 300 nM TAK-243 for 18 hours in the presence or absence of IFN-γ stimulation. **f**, Flow cytometry measuring surface expression of MHC-I in CT26 cells treated with 150 nM or 300 nM TAK-243 for 18 hours in the presence or absence of IFN-γ or IFN-β stimulation. IFN stimulation was performed for 18 hours, with IFN-γ at 1 ng/mL and IFN-β at 0.1 ng/mL. **g**, Mass spectrometry, shown in **Figure 5g**, with the proteins of interest being highlighted. LFC: Log_2 fold change. **h**, Representative images (left) and quantification (right) of Immunoblot analysis assessing levels of the indicated proteins in Myc-CaP cells treated with 50 nM or 100 nM TAK-243 for 7 or 14 hours (hr). Data were acquired from three biological replicates. Data are presented as mean ± SEM. Statistics were acquired by two-tailed Student's t test. **p*<0.05; ***p*<0.01; n.s.: not significant. **i**, Mass spectrometry, shown in **Figure 5g**, with the proteins of interest being highlighted.

Data are presented as mean ± SD in **a** and **f**, and mean ± SEM in **c, d** and **h**. Statistics were acquired by two-tailed Student's t test. Data in **a** and **f** were acquired from technical triplicates, representative of two independent experiments.
