# Supplementary material for: The UBA1–STUB1 Axis Mediates Cancer Immune Escape and Resistance to Checkpoint Blockade
Source: Cancer Discov. 2024 Nov 14;15(2):363–81. doi: 10.1158/2159-8290.CD-24-0435 (PMC11803397; doi:10.1158/2159-8290.CD-24-0435)
Supplement: Supplementary Figure S8 — UBA1 mediates JAK1 ubiquitination via STUB1. [file cd-24-0435_supplementary_figure_s8_suppsf8.pdf]

Supplementary Figure S8

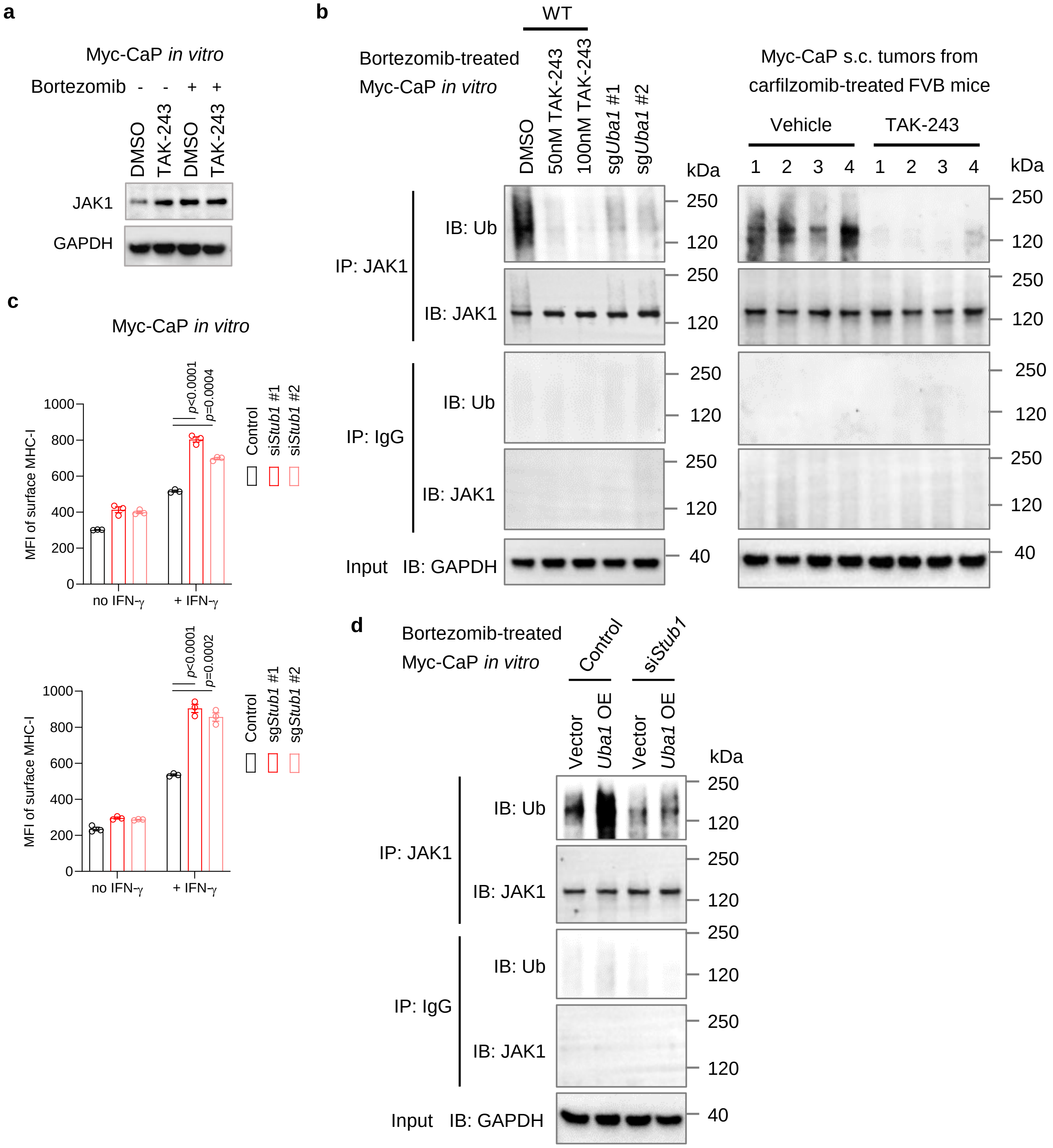

Supplementary Figure S8: a, Immunoblot analysis assessing levels of the indicated proteins in Myc-CaP cells treated with or without 75 nM TAK-243 for six hours and then with or without 1 µM bortezomib for an additional six hours. **b**, Immunoblot analysis assessing levels of the indicated proteins after immunoprecipitation with anti-JAK1 antibody or the control IgG in bortezomib-treated Myc-CaP cells (left) or in Myc-CaP subcutaneous (s.c.) tumors derived from carfilzomib-treated FVB mice (right). Ub: ubiquitin. **c**, Flow cytometry measuring surface MHC-I expression in Myc-CaP cells that received distinct siRNAs (top) or sgRNAs (bottom) targeting *Stub1*. Non-targeting siRNA or sgRNA was used as control, respectively. Data are presented as mean ± SD. Statistics were acquired by two-tailed Student's t test. **d**, Immunoblot analysis assessing levels of the indicated proteins after immunoprecipitation with anti-JAK1 antibody or the control IgG in the indicated bortezomib-treated Myc-CaP cells. OE: overexpression.
