# Supplementary material for: The UBA1–STUB1 Axis Mediates Cancer Immune Escape and Resistance to Checkpoint Blockade
Source: Cancer Discov. 2024 Nov 14;15(2):363–81. doi: 10.1158/2159-8290.CD-24-0435 (PMC11803397; doi:10.1158/2159-8290.CD-24-0435)
Supplement: Supplementary Figure S9 — UBA1 inactivation upregulates interferon signaling in vivo. [file cd-24-0435_supplementary_figure_s9_suppsf9.pdf]

Supplementary Figure S9

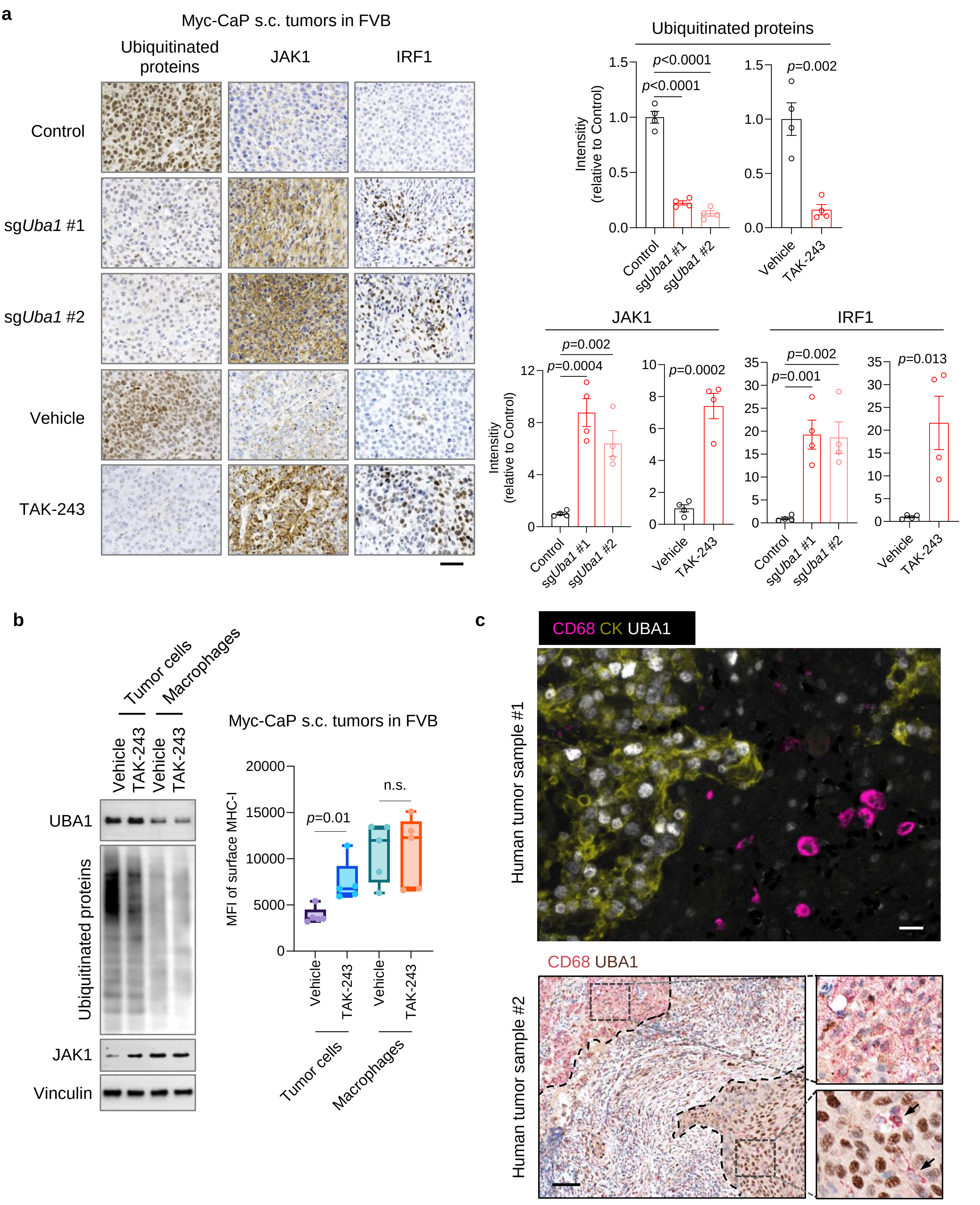

Supplementary Figure S9: a, Representative images (left) and quantification (right) of immunohistochemistry measuring levels of indicated proteins in *Uba1*-depleted or -inhibited Myc-CaP subcutaneous (s.c.) tumors from FVB mice. Scale bar: 50 µm. **b**, Immunoblot analysis assessing levels of the indicated proteins (left) and flow cytometry measuring expression of surface MHC-I (right) in tumor cells or intratumoral macrophages sorted from Myc-CaP subcutaneous (s.c.) tumors. These tumors were harvested 24 hours after a single dose of TAK-243 administration (*n* = 5 mice per group). Immunoblot analysis samples were pooled from five mice per group. **c**, Top: multiplex immunofluorescence staining CD68 (a macrophage marker), pan-cytokeratin (CK; a tumor cell marker), and UBA1 in a prostate cancer tumor. Scale bar: 20 µm. Bottom: Duplex immunohistochemistry staining CD68 and UBA1 in an independent prostate cancer tumor. A dashed line in the top left marks an area with a high abundance of macrophages. A dashed line in the bottom right marks a tumor area. Arrows point out intratumoral macrophages. Scale bar: 50 µm.

Data are presented as mean ± SEM in **a** or box and whisker plots in **b**. Statistics were acquired by two-tailed Student's t test.
