# Supplementary material for: The UBA1–STUB1 Axis Mediates Cancer Immune Escape and Resistance to Checkpoint Blockade
Source: Cancer Discov. 2024 Nov 14;15(2):363–81. doi: 10.1158/2159-8290.CD-24-0435 (PMC11803397; doi:10.1158/2159-8290.CD-24-0435)
Supplement: Supplementary Figure S10 — UBA1 inactivation upregulates interferon signaling in human cancer. [file cd-24-0435_supplementary_figure_s10_suppsf10.pdf]

Supplementary Figure S10

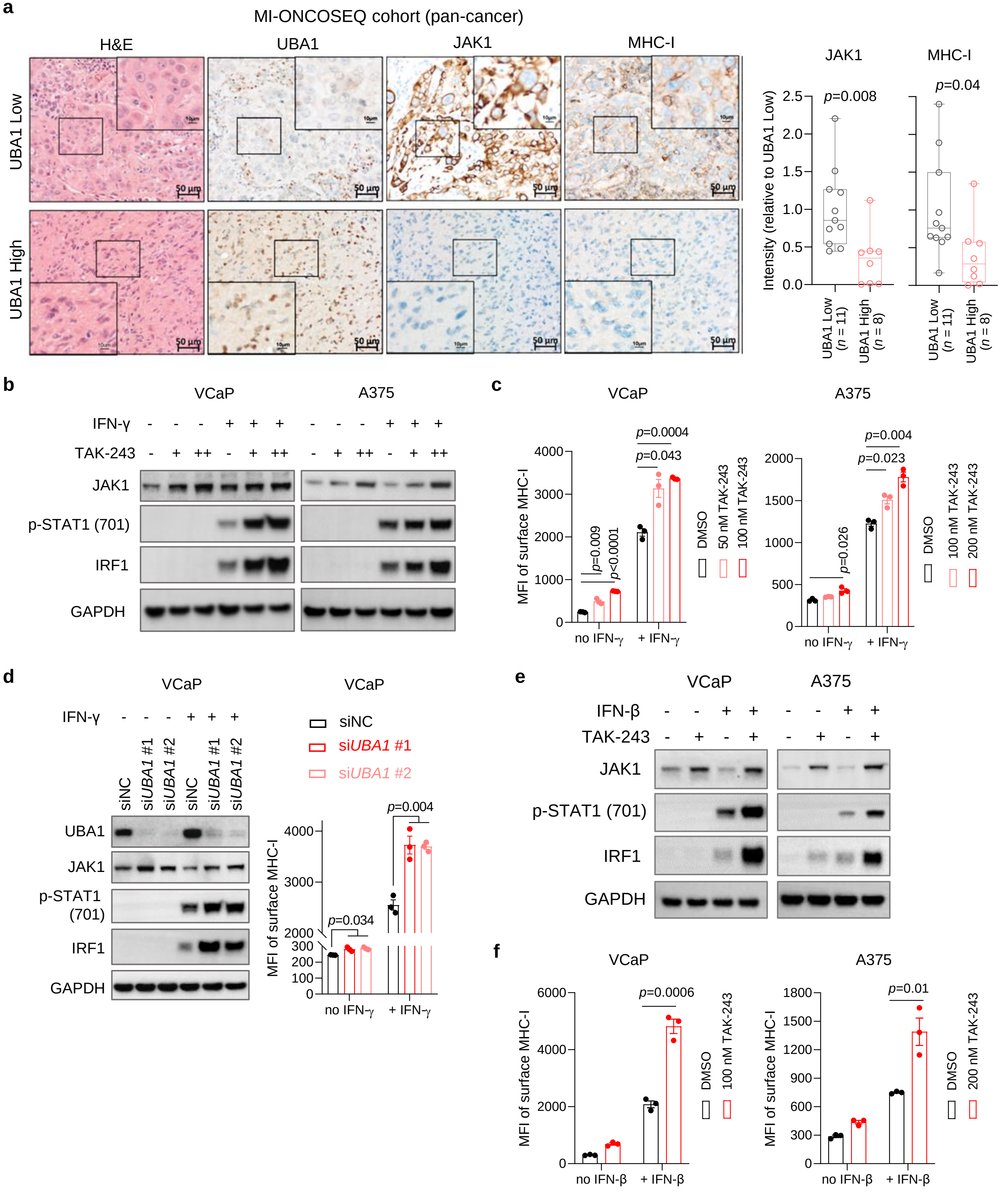

Supplementary Figure S10: a, Representative images (left) and quantification (right) of immunohistochemistry measuring levels of indicated proteins in tumors from a cohort treated at the University of Michigan, Ann Arbor (MI-ONCOSEQ cohort). Scale bar: 50 µm. Data are presented as box and whisker plots and statistics were acquired by two-tailed Student's t test. **b–c**, Immunoblot analysis assessing levels of the indicated proteins (**b**) and flow cytometry measuring surface expression of MHC-I (**c**) in indicated cells treated with low or high doses of TAK-243 for 18 hours in the presence or absence of 1 ng/mL IFN-γ stimulation. Concentrations of TAK-243 used in each cell line are indicated in **c**. **d**, Immunoblot analysis assessing levels of the indicated proteins (left) and flow cytometry measuring surface expression of MHC-I (right) in the VCaP cells transfected with independent siRNAs targeting *UBA1* (si*UBA1* #1 and si*UBA1* #2) or non-targeting siRNA (siNC) and treated with or without 1 ng/mL IFN-γ for 18 hours. **e–f**, Immunoblot analysis assessing levels of the indicated proteins (**e**) and flow cytometry measuring surface expression of MHC-I (**f**) in the indicated cancer cells treated with or without TAK-243, and stimulated with or without 0.1 ng/mL IFN-β for 18 hours.

Data are presented as mean ± SEM. Statistics were acquired by two-tailed Student's t test in **a**, **c**, and **f**, or two-way ANOVA in **d**. Data in **b** and **c** were acquired from technical triplicates, representative of two independent experiments.
